# Optimization of Operation Parameters for Helical Flow Cleanout with Supercritical CO_2_ in Horizontal Wells Using Back-Propagation Artificial Neural Network

**DOI:** 10.1371/journal.pone.0156358

**Published:** 2016-06-01

**Authors:** Xianzhi Song, Chi Peng, Gensheng Li, Zhenguo He, Haizhu Wang

**Affiliations:** 1 State Key Laboratory of Petroleum Resource and Prospecting, China University of Petroleum-Beijing, Changping, Beijing, China; 2 College of Petroleum Engineering, China University of Petroleum-Beijing, Beijing, China; Tianjin University, CHINA

## Abstract

Sand production and blockage are common during the drilling and production of horizontal oil and gas wells as a result of formation breakdown. The use of high-pressure rotating jets and annular helical flow is an effective way to enhance horizontal wellbore cleanout. In this paper, we propose the idea of using supercritical CO_2_ (SC-CO_2_) as washing fluid in water-sensitive formation. SC-CO_2_ is manifested to be effective in preventing formation damage and enhancing production rate as drilling fluid, which justifies tis potential in wellbore cleanout. In order to investigate the effectiveness of SC-CO_2_ helical flow cleanout, we perform the numerical study on the annular flow field, which significantly affects sand cleanout efficiency, of SC-CO_2_ jets in horizontal wellbore. Based on the field data, the geometry model and mathematical models were built. Then a numerical simulation of the annular helical flow field by SC-CO_2_ jets was accomplished. The influences of several key parameters were investigated, and SC-CO_2_ jets were compared to conventional water jets. The results show that flow rate, ambient temperature, jet temperature, and nozzle assemblies play the most important roles on wellbore flow field. Once the difference between ambient temperatures and jet temperatures is kept constant, the wellbore velocity distributions will not change. With increasing lateral nozzle size or decreasing rear/forward nozzle size, suspending ability of SC-CO_2_ flow improves obviously. A back-propagation artificial neural network (BP-ANN) was successfully employed to match the operation parameters and SC-CO_2_ flow velocities. A comprehensive model was achieved to optimize the operation parameters according to two strategies: cost-saving strategy and local optimal strategy. This paper can help to understand the distinct characteristics of SC-CO_2_ flow. And it is the first time that the BP-ANN is introduced to analyze the flow field during wellbore cleanout in horizontal wells.

## 1. Introduction

Sand production is the result of formation breakdown. In horizontal wells, unexpected formation breakdown occurs when 1) the drilling fluid pressure exceeds the fracture pressure of formation; 2) the flowing bottom hole pressure is too low (or equivalently, the flow rate is too high) during production. Well cleanout shall be done when sand production is severe. Water jets are used extensively in conventional horizontal wellbore cleanouts to break up consolidated sand deposits and sweep away solids. In horizontal wells, the flow direction is perpendicular to the gravity, so the sands have a tendency to settle down, resulting in low cleanout efficiency or even worse troubles such as stuck-pipe [1]. Over the past years, several improved cleanout techniques, such as wiper tripping and sand vacuuming [2], have been developed. Among these techniques, the helical flow approach, which applies partial high-pressure jets to generate spiral flow in annulus, has been verified to be efficient by simulations and field applications [3–6]. Compared to the conventional method, the annular helical flow approach has better performance at suspending and transporting solids [7–8]. Experiments have been conducted to illustrate the influences of operational parameters, including fluid properties, pumping rate, and borehole diameter, on cleanout efficiency in inclined wells [9–12]. Song et al. analyzed the mechanism and characteristics of helical flow in horizontal well cleanout with water-based fluids [2].

Since the last decade, SC-CO_2_ has been regarded as a promising new drilling fluid in the oil and gas industry [13,14,16,17]. It has some preeminent properties, including a liquid-like density, a low viscosity close to gas and a good diffusion coefficient [13–18], which enables it to outperform traditional foam and nitrogen fluid in underbalanced drilling. Several models were developed to calculate the density and viscosity of SC-CO_2_ both in drilling strings and annuls based on ambient pressure, temperature and depth, as well as friction loss and jet impact [13,19]. Field application was also studied to justify the feasibility of SO-CO_2_ drilling [20].

Although there is few applications of SC-CO_2_ cleanout thus far, SC-CO_2_ is considered to be more suitable than conventional flushing fluid in water-sensitive, low-permeability, and unconventional reservoirs owing to the advantages of minor damage to the hydrocarbon formations [21]. When performing cleanout, wellbore pressure is the decisive factor of formation damage. If the wellbore pressure is lower than formation pressure, formation damage due to flushing fluid invasion shall never happen. In this paper the over-pressure situation is postulated. The advantage of using SC-CO_2_ under over-pressure condition is that carbon dioxide will cause less damage compared with water. The general mechanism of formation damage in water-sensitive, water-wet formation goes like this: firstly, water-based flushing fluid is filtered into formation next to the wellbore as a result of the positive pressure gradient from wellbore to formation. Then water induces damage by: 1) causing the expansion, dispersion and transportation of clay (if any), which can block throats or pores; 2) blocking microchannel due to capillary effect. Both phenomena will increase the skin factor and decrease permeability. On the other hand, in SC-CO_2_ washing, the only thing that can filter into formation is carbon dioxide, which does not interact with clay and is miscible with oil. Thus, there will be no blocking and consequently much less damage.

Computational fluid dynamic (CFD) simulation is a reliable method to investigate a complex flow field comprehensively [22,23]. In this paper, the CFD method was used to simulate the helical flow field in a horizontal wellbore created by directional high-pressure jets. However, only one set of parameters can be analyzed in a single CFD simulation. In order to optimize the cleanout operation parameters, the complex relationship between parameters and flow field velocities need to be displayed in such a way that velocities can be output directly from input parameters. In other words, data analysis and fitting should be carried out.

There are many intelligence methods that can be applied in data fitting: Genetic Algorithm (GA) that simulates natural selection and heredity process, Simulated Annealing Algorithm (SAA) that simulates the stochastic state of solid particles cooling from high temperature, Artificial Immune Algorithm (AIA) that is based on the biological immunity theory, Artificial Neural Network (ANN) that simulates the signal transition between neurons, Support Vector Machine (SVM) et al. Each method has its strength and weakness. For example, ANN is very efficient at data fitting but over-fit may happen. SVM is a simple but robust tool for data fitting, especially for small sample and non-linear problems. But it becomes less efficient with large data size [24,25].

There are some recent progresses in fluid flow analysis using complex network that need to be addressed. Complex network has enjoyed a noteworthy development in the last decade and it provides a useful framework to investigate complex systems (in this case, complex fluid flows) from different perspectives [26–30]. To build a complex network from a complex system, system components (flow signals) are represented as nodes while the interactions between nodes are regarded as edges. In a real complex network, there are ‘community’ structures that incorporate certain group of nudes. Nodes are compactly interconnected within a community and the links between communities are sparse. Community detection is of great significance for clarifying the structure of complex networks. A reliable method for constructing directed weighted complex network from time series data was established in [26]. The method introduced was a faithful tool to extract the dynamical information from experimental signals in complex systems. In order to uncover the transitional behavior of slug flow, Gao et al. [27] calculated multivariate pseudo Wigner distribution (MPWD) and the multivariate multiscale sample entropy (MMSE) for different flow conditions. The results indicated combining the MPWD and MMSE enabled to reveal the transient and multiscale flow behavior from slug flow to churn flow. What’s more, charactering the dynamic behavior and flow structure of two-phase flow, which is a challenging problem of significant industrial importance, can be accomplished using complex network. Complex networks with various properties corresponding to different situations have been successfully built to investigate this issue. In experimental horizontal oil-water two-phase flow, a complex network-based method was proposed to distinguish complex flow patterns [28]. The results suggested that the community detection in two-phase flow complex network enabled to objectively distinguish intricate horizontal oil-water flow patterns, while the conventional method based on adaptive optimal kernel time-frequency representation (AOK TFR) was invalid. Studies on similar problems have been accomplished with methods based on complex network: the transitions and nonlinear dynamic behaviors of gas-liquid flow patterns were quantitatively uncovered based on the distinct topological structures of multivariate complex networks derived from different flow conditions [29]. A new approach based on multi-frequency complex network was proposed to uncover the horizontal oil-water flow structures from experimental multivariate measurements [30]. It was revealed that the community structures could robustly represent the structural features of different flow patterns. In [31], the vertical upward oil-water two-phase flow, which was a multiscale, unstable and non-homogenous complex system, was analyzed using a multivariate multiscale complex network. The results suggested that the clustering coefficient entropy from complex network could not only indicate the oil-in-water flow pattern transition but also show the dynamic behavior of vertical two-phase flow. Based on these applications, the ability of complex network on data analysis of two-phase flow has been fully manifested and it is believed complex network is highly potential to be applied in other fluid flow analysis.

To effectively analyze and fit the data, the BP-ANN was employed in this paper. BP-ANN has been applied with great adaptation and generalization to many other applications in petroleum engineering. Yu et al. [32] combined an ANN with the genetic algorithm and simulated annealing to predict oil reserve quantities based on geological data. Wang et al. built a mathematical model of the main dimensions for self-elevating drilling units by means of BP-ANN [33]. Kaydani et al. and Irani et al. applied an improved ANN to fit core permeability with well logging and geological data [34,35]. The prediction of permeability in a homogeneous area can also be achieved by an ANN with other intelligence algorithms [36]. The bottom hole pressure was predicted precisely without flow pattern determination in underbalanced drilling by applying an ANN [37]. Wang et al. made a 9-variable model based on an ANN to select appropriate deepwater floating platforms [38]. Peng et al. predicted the working life of coiled tubing based on the BP algorithm of an ANN [39]. El-Abbasy et al. developed an experience-based neural network model to predict the rate of failure and working conditions for oil and gas pipelines [40].

Previous cases proved that an ANN shows excellent accuracy when disposing of fitting, regression and prediction problems. Thus, it can be a feasible method for analyzing simulation data and predicting flow field features. On the other hand, SVM also seems to be a qualified method and there have been many comparison studies between ANN and SVM. Though it seems that in most cases SVM has better performance than ANN [41–47], there are some cases vice versa [48]. Besides, they can also have similar accuracy under certain circumstance [49,50]. Thus, in this paper SVM (more specifically, Support Vector Regression, SVR) was also applied to be compared with BP-ANN in terms of efficiency and accuracy.

The objectives of this paper include are as follows: 1) simulating the SC-CO_2_ helical flow field during cleanout; 2) investigating the influence of operation parameters on the SC-CO_2_ helical flow field; 3) comparing the differences between SC-CO_2_ helical flow and water flow; 4) and optimizing the operation parameters employing BP-ANN approach based on the cost-saving strategy (CSS) and local optimal strategy (LOS).

## 2. Material and Methods

### 2.1 Simulation of SO-CO_2_ helical flow cleanout

#### 2.1.1 Wellbore model

**Fig 1:**
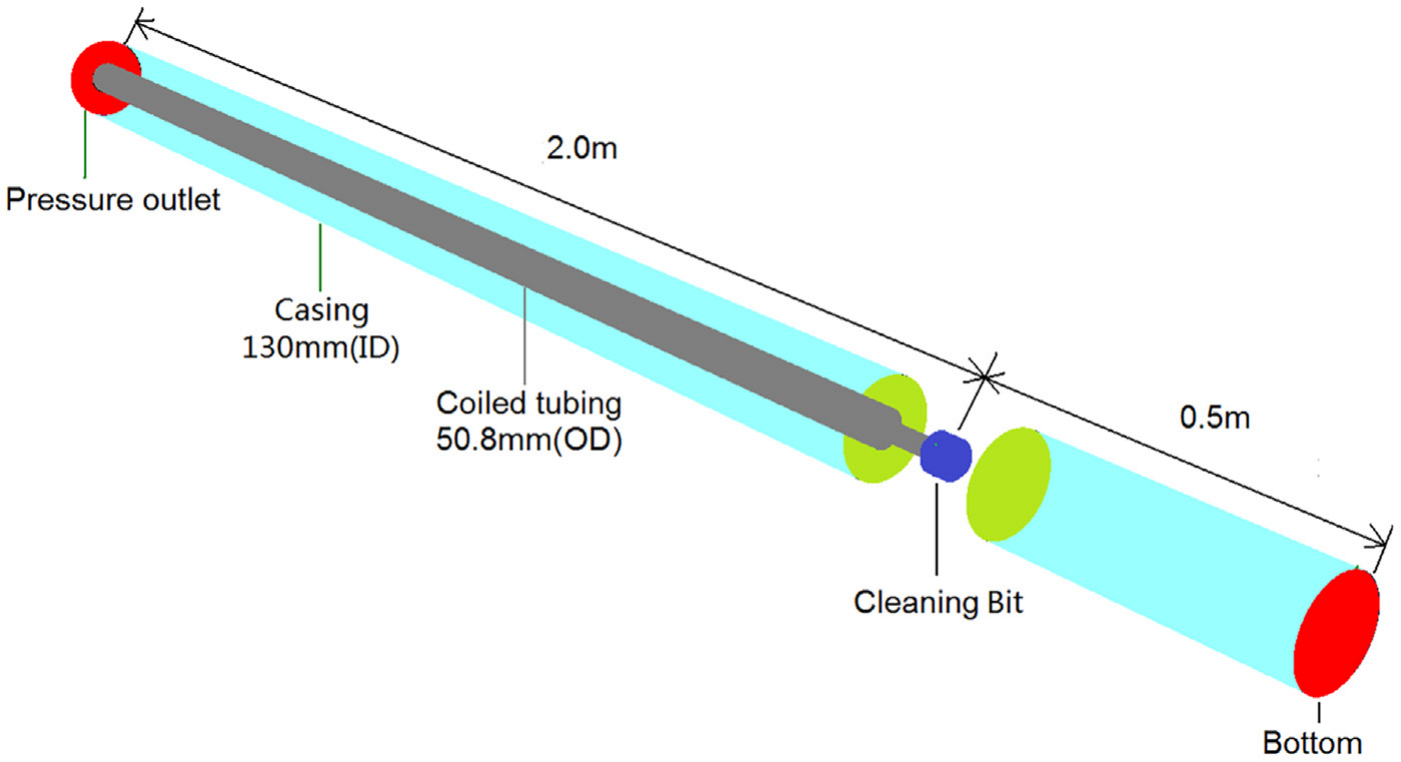
The wellbore model of helical flow cleanout in horizontal wells.

**Fig 2:**
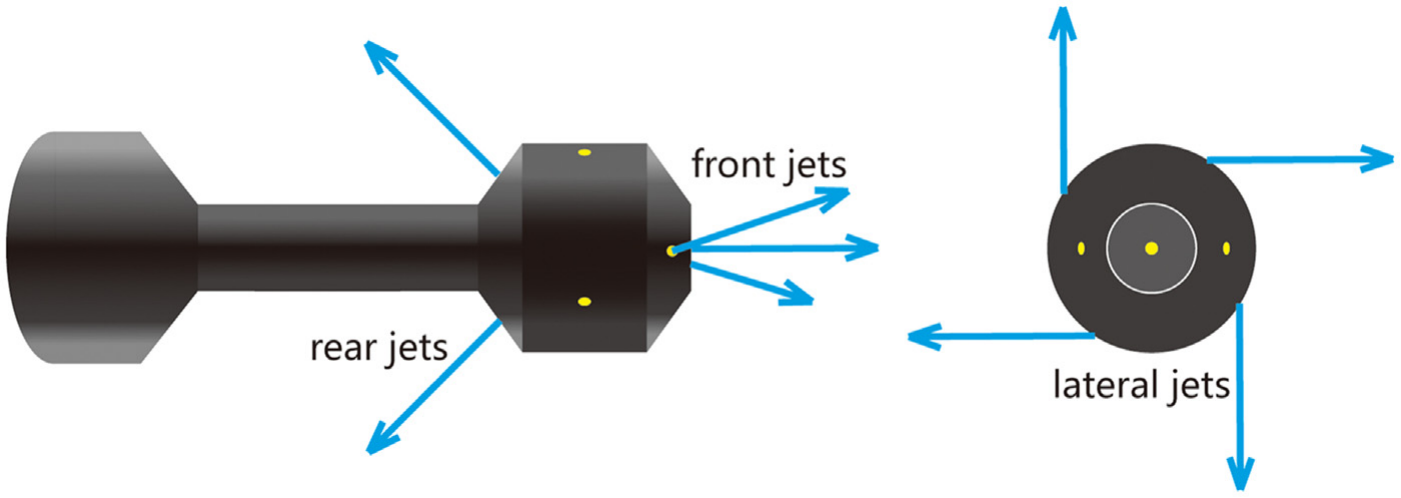
Nozzle assembly and jet orientations of the cleanout bit.

Fig 1 shows a wellbore model with a typical helical flow cleanout tool [2], with 130 mm (inner diameter) casing and 50.8 mm (outer diameter) coiled tubing. As shown in Fig 2, nine nozzles are assembled on the jetter, grouped as forward nozzles, lateral nozzles and rear nozzles. Each group of nozzles can produce high-pressure SC-CO_2_ jets during circulation and has specific functions. Impinging forward, the forward jets are designed to break up encountered consolidated deposits into floating particles. The lateral jets are not orthogonal to the casing. Instead, they impact the casing at predefined angles, thus generating a strong helical flow in the annulus. The rear jets can improve the sweeping efficiency by pushing floating solids backward.

**Fig 3:**
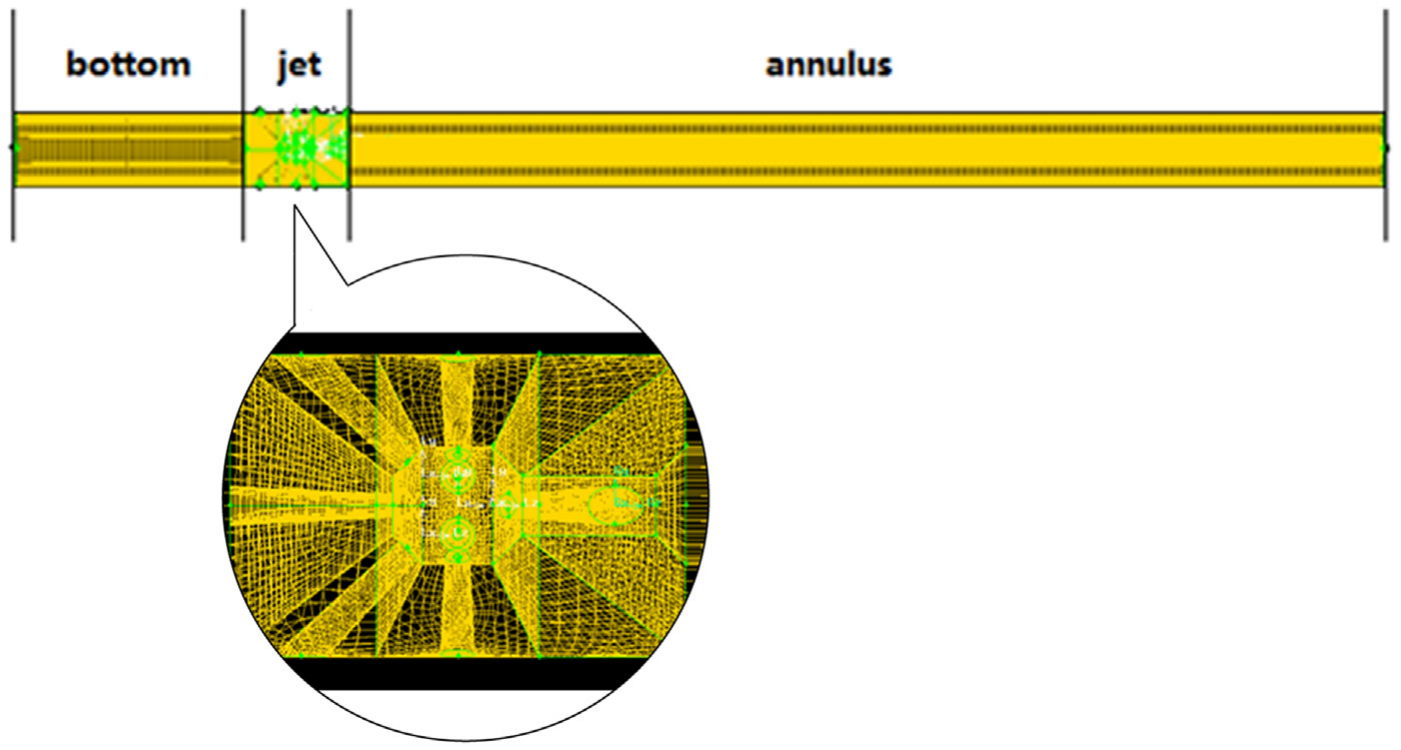
The meshed wellbore model. The mesh near the nozzle exits are deliberately refined so that convergence can be accelerated and more precise simulation of the flow field can be achieved.

The concentric annulus and well bottom are regular geometries where flow field is relatively stable, so they were meshed by structural grids. In contrast, the flow field is much chaotic near the exit regions of nozzles. Based on this fact, the whole model was divided and meshed in three sections, as displayed in Fig 3. Particularly, to characterize the flow field precisely and accelerate convergence, nine infill frustum grids were added along the initial jet paths. Corresponding boundary types of walls, velocity inlet, pressure outlet and interfaces were defined.

#### 2.1.2 Mathematical models

*Governing equations*.

Continuity equation

$$\frac{\partial \rho }{\partial t}+\frac{\partial \rho v_{x}}{\partial x}+\frac{\partial \rho v_{y}}{\partial y}+\frac{\partial \rho v_{z}}{\partial z}=0$$ (1)

where *v*_*i*_ (*i* = *x*, *y*, *z*) is the velocity component in the *i* direction; *ρ* is fluid density; *t* is time.

Momentum equation

$$\begin{array}{l} \frac{\partial \rho v_{x}}{\partial t}+\sum_{i}\frac{\partial \rho v_{i}v_{x}}{\partial x_{i}}=\rho g_{x}-\frac{\partial P}{\partial x}+R_{x}+T_{x}+\sum_{i}\frac{\partial }{\partial x_{i}}\left(\mu_{e}\frac{\partial v_{x}}{\partial x_{i}}\right)\\ \frac{\partial \rho v_{y}}{\partial t}+\sum_{i}\frac{\partial \rho v_{i}v_{y}}{\partial x_{i}}=\rho g_{y}-\frac{\partial P}{\partial y}+R_{y}+T_{y}+\sum_{i}\frac{\partial }{\partial x_{i}}\left(\mu_{e}\frac{\partial v_{y}}{\partial x_{i}}\right)\\ \frac{\partial \rho v_{z}}{\partial t}+\sum_{i}\frac{\partial \rho v_{i}v_{z}}{\partial x_{i}}=\rho g_{z}-\frac{\partial P}{\partial x}+R_{z}+T_{z}+\sum_{i}\frac{\partial }{\partial x_{i}}\left(\mu_{e}\frac{\partial v_{z}}{\partial x_{i}}\right) \end{array}$$ (2)

where *g*_*i*_ is the component of gravity acceleration in *i* direction; ∂*x*_*i*_ is the partial differential in *i* direction; *μ*_*e*_ is effective viscosity; *R*_*i*_ is distributed resistance in *i* direction; *T*_*i*_ is viscous loss term in *i* direction.

Energy equation

$$\frac{\partial }{\partial t}\left(\rho C_{p}T_{0}\right)+\sum_{i}\frac{\partial }{\partial x_{i}}\left(\rho v_{i}C_{p}T_{0}\right)=\sum_{i}\frac{\partial }{\partial x_{i}}\left(K\frac{\partial T_{0}}{\partial x_{i}}\right)+W^{v}+E^{k}+Q_{v}+\phi +\frac{\partial P}{\partial t}$$ (3)

where *C*_*p*_ is specific heat; *T*_*0*_ is total (or stagnation) temperature; *K* is thermal conductivity; *W*^*v*^ is the viscous work term; *Q*^*v*^ is the volumetric heat source; Φ is the viscous heat generation term; *E*^*k*^ is kinetic energy;

*Turbulence model*. According to the helical characteristics of the flow field, the RNG *k*−*ε* model was chosen to describe the flow field for better performance [6,51], which has a similar form to the standard *k*−*ε* model:

$$\frac{\partial }{\partial t}\left(\rho k\right)+\frac{\partial }{\partial x_{i}}\left(\rho kv_{i}\right)=\frac{\partial }{\partial x_{j}}\left(\alpha_{k}\mu_{eff}\frac{\partial k}{\partial x_{j}}\right)+G_{k}+G_{b}-\rho \varepsilon -Y_{M}+S_{k}$$ (4)

$$\frac{\partial }{\partial t}\left(\rho \varepsilon \right)+\frac{\partial }{\partial x_{i}}\left(\rho \varepsilon v_{i}\right)=\frac{\partial }{\partial x_{j}}\left(\alpha_{\varepsilon }\mu_{eff}\frac{\partial k}{\partial x_{j}}\right)+G_{1\varepsilon }\frac{\varepsilon }{k}\left(G_{k}+C_{3\varepsilon }G_{b}\right)-2G_{2\varepsilon }\rho \frac{\varepsilon^{2}}{k}-R_{\varepsilon }+S_{\varepsilon }\;$$ (5)

where *G*_*k*_ is the generation of turbulence kinetic energy due to the mean velocity gradients; *G*_*b*_ is the generation of turbulence kinetic energy due to buoyancy; *Y*_*M*_ is the contribution of the fluctuating dilatation in compressible turbulence to the overall dissipation rate; *α*_*k*_ and *α*_*ε*_ are the inverse effective Prandtl numbers for *k* and *ε*; *S*_*k*_ and *S*_*ε*_ are source terms.

*Sate equations*. When flowing in annulus, the properties of SC-CO_2_ are controlled by temperature and pressure. Thus, the state equations of carbon dioxide should also be considered. In the numerical simulation, the state equation developed by Span and Wagner [52], which is recommended by NIST, was used to calculate the density and isobaric heat capacity of SC-CO_2_. Based on Helmholtz energy, Span-Wagner state equation is a highly accurate reference equation for the thermodynamic properties of pure carbon dioxide. It can be applied with temperature ranging from the triple-point temperature to 1000 K and a maximum pressure of 2200 MPa.

The Helmholtz energy (A) is the function of two independent variables: density (ρ) and temperature (T), namely, A = A(ρ, T). Span-Wagner state equation is a function of dimensionless Helmholtz energy (α), which is given as follows [52]:

$$\alpha \left(\delta ,\tau \right)=\frac{A\left(\rho ,T\right)}{RT}$$ (6)

where *δ* is dimensionless density (*δ* = *ρ*/*ρ*_*c*_); *τ* is inverse dimensionless temperature (*τ* = *T*_*c*_/*T*); *ρ* is the density of CO_2_, kg/m^3^; *T* is temperature, K; *R* is the gas constant, kJ/ (kg∙K); *T*_*c*_ is the critical temperature of CO_2_, K; *ρ*_*c*_ is the critical density of CO_2_, kg/m^3^.

The dimensionless Helmholtz energy is composed of two parts:

$$\alpha (\delta ,\tau )=\alpha^{o}(\delta ,\tau )+\alpha^{r}(\delta ,\tau )$$ (7)

where *α*^*o*^ is the ideal part of dimensionless Helmholtz energy; *α*^*r*^ is the residual part.

The density and isobaric heat capacity (*C*_*p*_) of CO_2_ are calculated by equations as follows:

$$\rho =\frac{p\;\left(\delta \text{, }\tau \right)}{R\;T\;\left(\text{ 1 }+\;\delta \;{\left(\frac{\partial \alpha^{r}}{\partial \delta }\right)}_{\tau }\right)}\;$$ (8)

$$C_{P}\text{(}\delta \text{,}\tau \text{)}=R\left[-\tau^{\text{2}}\left(\frac{\partial^{2}\alpha^{o}}{\partial \tau^{2}}+\frac{\partial^{2}\alpha^{r}}{\partial \tau^{2}}\right)\;+\;\frac{{(1\;+\;\delta \frac{\partial \alpha^{r}}{\partial \delta }-\delta \;\tau \;\frac{\partial^{2}\alpha^{r}}{\partial \delta \;\partial \tau }\text{)}}^{\text{2}}}{\text{1}+\text{2}\delta \frac{\partial \alpha^{r}}{\partial \delta }+\delta^{2}\;\frac{\partial^{2}\alpha^{r}}{\partial \delta^{2}}}\right]$$ (9)

where *p* is pressure, kPa.

In addition, the viscosity and thermal conductivity of CO_2_ are calculated using the model presented by Fenghour et al, recommended by NIST [53,54]. In this model, viscosity (η) is divided into two different terms as in Eq 10:

$$\eta \left(\rho ,T\right)=\eta_{0}\left(T\right)+\eta_{R}\left(\tau ,\delta \right)$$ (10)

where *η* is the viscosity of CO_2_, Pa∙s; *η*_*0*_ is the viscosity of dilute gas, Pa∙s; *η*_*R*_ is the residual part of viscosity, Pa∙s.

The thermal conductivity (*λ*) is calculated in a similar manner:

$$\lambda \left(\rho ,T\right)=\lambda_{0}\left(T\right)+\lambda_{R}\left(\tau ,\delta \right)+\lambda_{c}\left(\tau ,\delta \right)$$ (11)

where *λ* is the thermal conductivity of CO_2_, W/ (m∙K); *λ*_*0*_ is the thermal conductivity of dilute gas, W/ (m∙K); *λ*_*R*_ is the residual part of thermal conductivity, W/(m∙K); *λ*_*c*_ is the critical enhancement of thermal conductivity, W/ (m∙K).

#### 2.1.3 Simulation parameters

Five main factors influencing the helical flow field were investigated: nozzle assembly, flow rate, ambient pressure, ambient temperature and jet temperature, as shown in Tables 1 and 2. Ten possible assemblies were applied in the simulation with flow rates ranging from 10 L/s to 20 L/s. The ambient pressure/temperature indicates the true vertical depth (TVD) of the well. In this paper, the ambient pressure changes from 10 MPa to 50 MPa, roughly corresponding to a TVD variation from 1000 m to 5000 m. The jet temperature changes from 353 K to 433 K, and the ambient temperature changes from 333 K to 433 K. Both temperature and pressure are determinants of the physical properties of SC-CO_2_. The leakage of CO_2_ into the formation is ignored.

**Table 1:** Orifice Diameters of Ten Nozzle Assemblies.

Nozzle diameters, mm	No.1	No.2	No.3	No.4	No.5	No.6	No.7	No.8	No.9	No.10
**Rear nozzles**	3	4	4	4	4	4	4	4	5	6
**Lateral nozzles**	4	3	4	4	4	4	5	6	4	4
**Forward nozzles**	4	4	3	4	5	6	4	4	4	4
**Forward middle nozzle**	5	5	5	5	5	5	5	5	5	5

The total area of nozzles decides the exit velocity of fluid, larger area means smaller exit velocity. The enlargement of a specific type of nozzle will increase its share of total flow rate but decrease the exit velocity for all nozzles since total nozzle area is increased.

**Table 2:** Data Set: Simulation Conditions.

Parameter	Nozzle assembly	Flow rate	Ambient pressure	Ambient temperature	Jet temperature
**Upper boundary**	No.10	20 L/s	50 MPa	433 K	433 K
**Lower boundary**	No.1	10 L/s	10 MPa	333 K	353 K

The simulations cover all the ten potential nozzle assemblies and a large range of parameters in practice.

#### 2.1.4 Simulation setup

**Fig 4:**
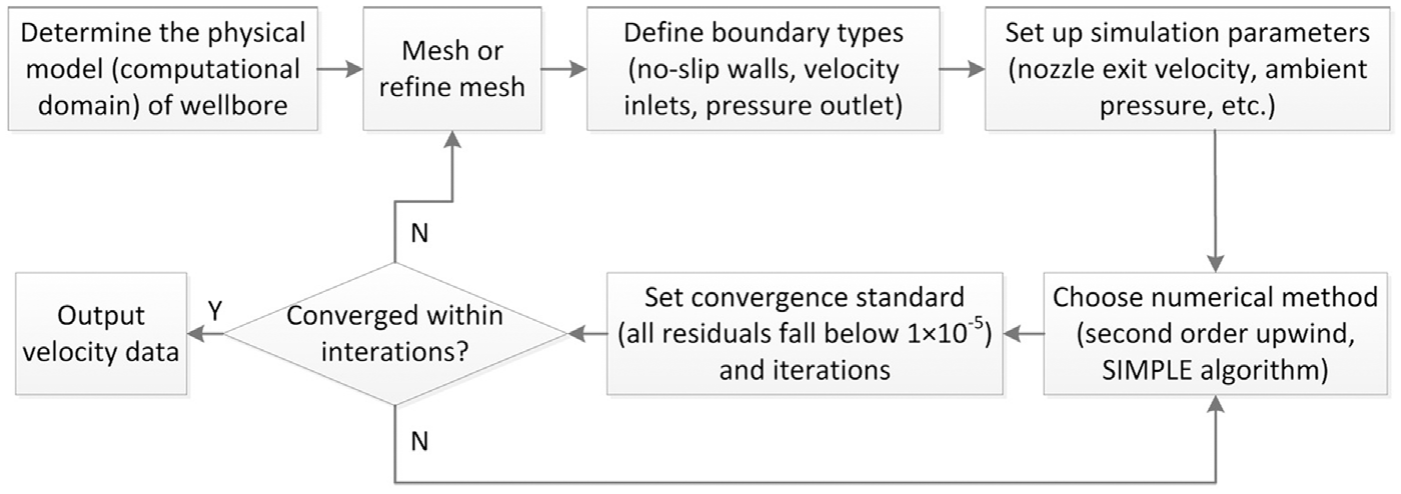
Numerical simulation process.

The steady flow situation was defined in all simulations. Governing equations were discretized using second order upwind scheme. The SIMPLE coupling method was used to solve the equations. To improve computation accuracy, the criterion of convergence was set as that all residuals fell blew 1×10^−5^ instead of default value 1×10^−3^. The simulations were accomplished using commercial CFD software ANSYS Fluent 14.5. A diagram depicting the whole simulation process is provided as Fig 4. It should be pointed out that when computation is not converged refining mesh and/or changing numerical method will be helpful to improve convergence.

### 2.2 BP-ANN Model

A sophisticated artificial intelligence algorithm, a BP-ANN, is basically a large class of parallel processing structures that are able to simulate vague and complicated connections between inputs and target data through the application of many nonlinear processing units called ‘neurons’ [55,56]. The connection between inputs and target data can be ‘learned’ by the neural network after adequate training [57,58]. A three-layer feed-forward neural network with a back-propagation algorithm can map any nonlinear relationship [59]. A potential problem that should be noted when using this powerful nonlinear regression method is over-fit. It is possible that there are some unqualified samples in the data. Over-fit is the match between the erroneous inputs and target data that introduces wrong relationships into the model. It will misunderstand ‘noise data’ as correct ones, thus impairing the certainty and precision of the connections between inputs and targets.

**Fig 5:**
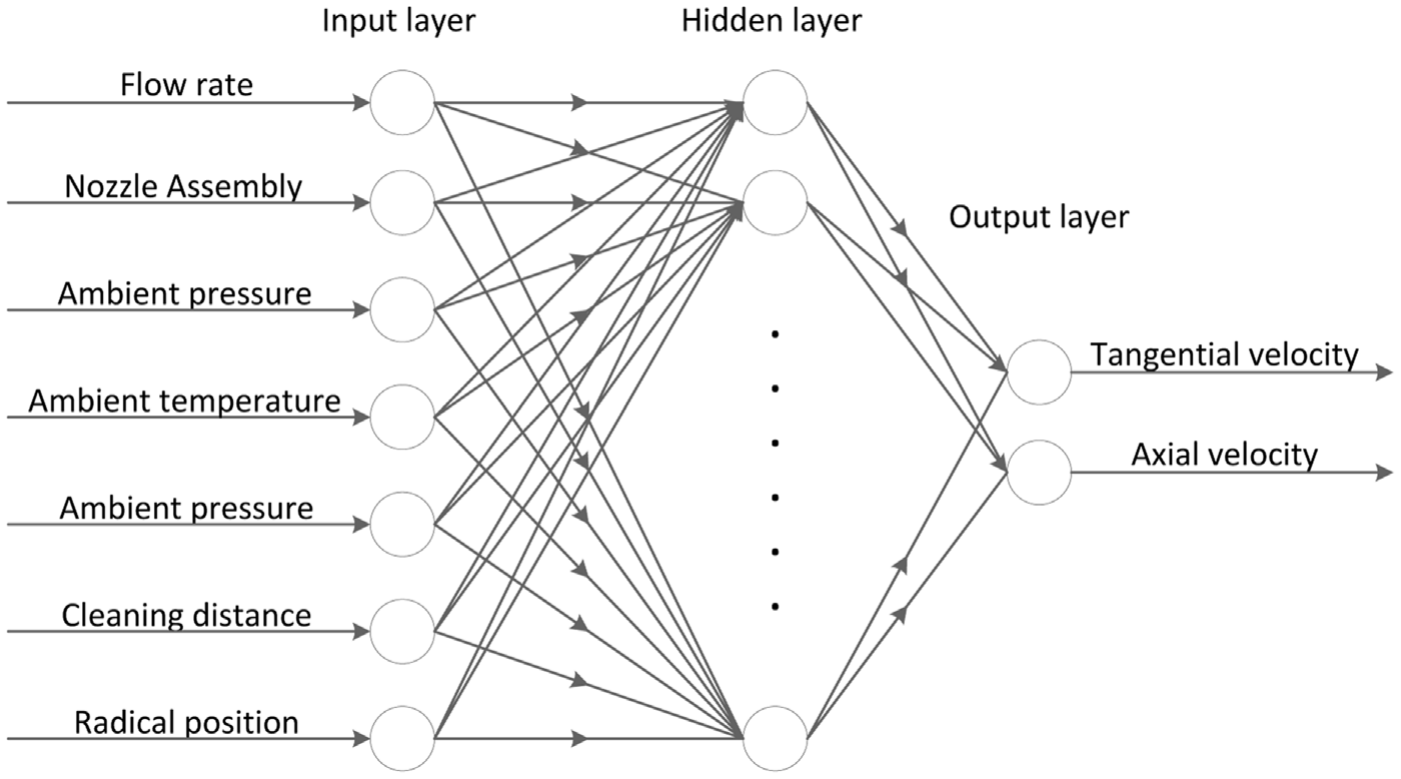
The topology of three-layer BP-ANN. This three-layer BP-ANN has 7 inputs (operation parameters) and 2 outputs (characteristic velocity V_t_ and V_a_). The transfer functions in the hidden layer are sigmoid. And the number of neurons in the hidden layer is 10.

In this paper, the tangential velocity (V_t_) and the annular velocity (V_a_) of a SC-CO_2_ helical flow field are set to be functions of flow rate, nozzle assembly, ambient pressure, ambient temperature, jet temperature, cleaning distance and radial position (dimensionless radius). A three-layer BP-ANN (Fig 5) is developed to match the operation parameters with V_t_ and V_a_, in which the transfer functions in the hidden layer are sigmoid, whereas those in the output layer are linear functions. The input layer consists of 7 neurons that represent the 7 operation parameters affecting the helical flow field, whereas the output layer has 2 neurons that represent V_t_ and V_a_. The number of neurons in the hidden layer determines how well a problem can be learned. If there are too few neurons, the network will be more generalized than necessary and fail to learn the specific patterns very well. Otherwise, if there are too many neurons, the network will have the tendency to memorize the specific problem and not be generalized enough for application [51]. In other words, the number of neurons in the hidden layer relies on the nature of the problem and available data. Neither too many nor too few neurons in the hidden layer should be used. The neuron number in hidden layer was set as 10 as the result of considerations of accuracy, training time and the risk of over-fit. To determine the optimal number of neurons in hidden layer, firstly we found a rough range of reasonable number of neurons based on references where the problems are nonlinear and have similar data structures (5~8 inputs, 1~2 outputs compared with 7 inputs and 2 outputs in this paper) [32,34–36,38,40]. Thus, the test range of neuron number was decided as 3 to 14. Then we tried each number and obtained corresponding training time and accuracy. When neuron number was small (3 to 6), convergence was fast but the accuracy was not satisfactory (R^2^ were below 0.96). With the increase of neuron number (7 to 11), BP-ANN became more accurate (R^2^ were above 0.98) but need more training time. An interesting phenomenon is that after neuron number was raised to 12, the times for iteration and single convergence dramatically increased. In this case over-fit was assumed to occur. So we narrowed down the range of neuron number as 7 to 11. In these range 10-neurons was chosen because it had the second highest accuracy and 11-neurons case with the highest accuracy was right at the edge of over-fit. There may be some deviation of the optimal neuron number. However it is revealed in the figure that the accuracy of BP-ANN model will only have tiny changes if the number of neurons varies a little.

Therefore, after several trials, we found that the best number of neurons in the hidden layer is found to be 10. In this way, a BP-ANN with a 7-10-2 topology is established (Fig 5).

During the training process of BP-ANN, the error is subsequently backward propagated through the network to modify the model parameters, such as the weights of different layers and neuron thresholds, by means of the gradient descent method. The purpose is to minimize the sum of the mean squared error (MSE) between network outputs and the target data. In this case, the target data are the real values of V_t_ and V_a_ from CFD simulations.

The MSE is given by:

$$U=\frac{1}{2}\sum_{i}^{M}\sum_{j}^{N}{\left[T_{j}(i)-Y_{j}(i)\right]}^{2}$$ (12)

where *U* is the sum of the MSE between the target data and the network outputs, *N* is the number of input samples, *M* is the number of output neurons, *T*_*j*_(*i*) is the *i*th component target value corresponding to the *j*th input, and *Y*_*j*_(*i*) is the network output that approximates the target value *T*_*j*_(*i*).

In this model, to avoid over-fit, all data are divided into three sets: training dataset, validation dataset and test dataset. During the learning process, the model is only fed the training data. The testing dataset is used to test the network during training and also to correct it continuously by adjusting the parameters (weights, thresholds, etc.). The validation dataset, which is not presented to the network during training, is used to validate the model. The model will be retrained if the validation performance is poor. To perform data classification, each dataset is randomly selected from the entire database according to the classification percentages. In this paper, the percentages of training, validation, and test datasets are fixed to be 70%, 15%, and 15%, respectively.

**Fig 6:**
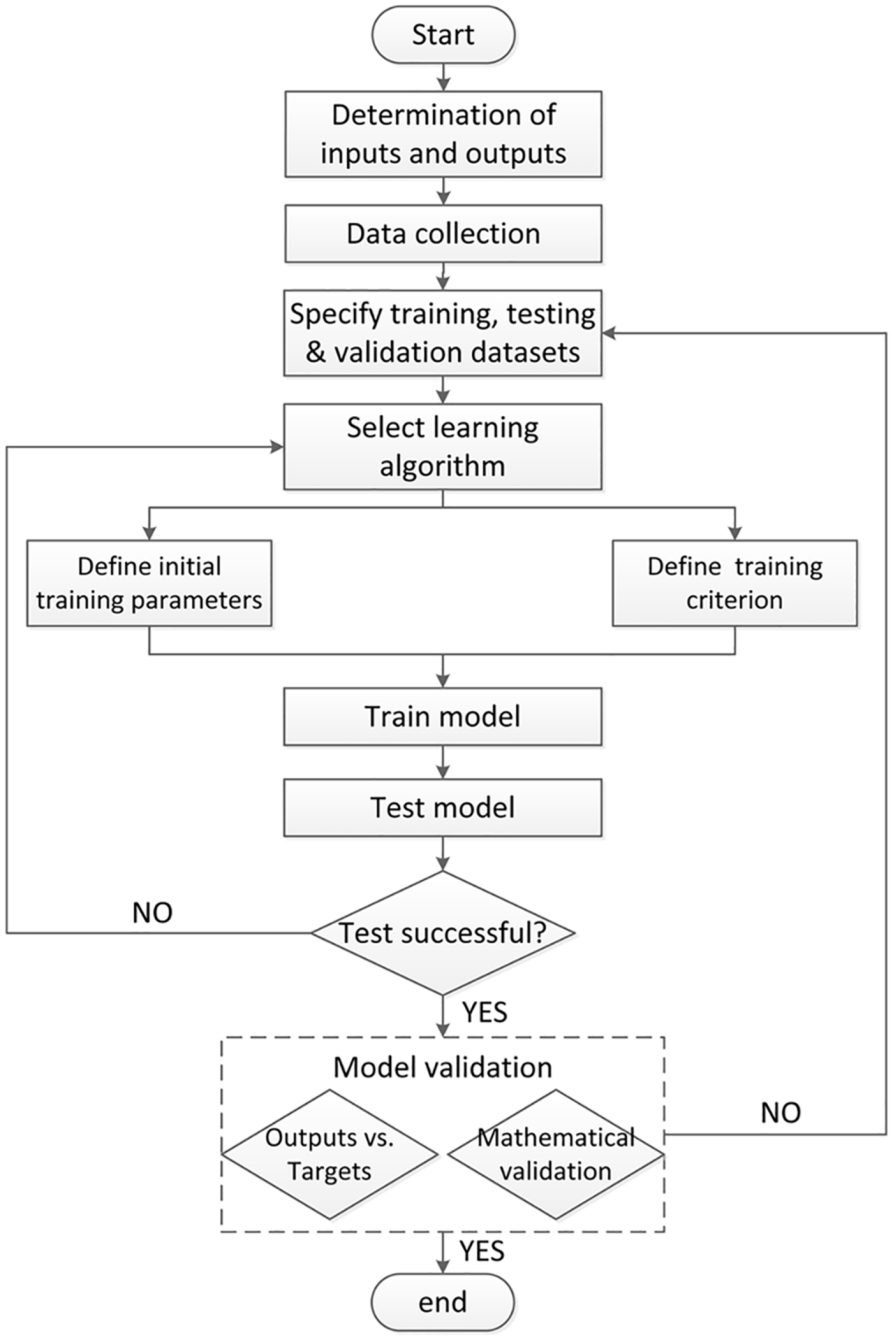
Framework of BP-ANN model. This process is accomplished in MATLAB.

The model framework is shown in Fig 6. Training iteration will end if a certain number of training epochs are conducted without a further decrease of absolute error between outputs and target data. When this criterion is met, training is stopped. If the validation performance is bad, the transfer functions or the number of neurons in the hidden layer should be changed.

## 3. Results and Discussion

### 3.1 Characteristics of the SC-CO_2_ helical flow field

In this part, the outcomes of the CFD simulation are analyzed mainly in terms of two major parameters of the SC-CO_2_ helical flow field—i.e., both tangential velocity (V_t_) and axial velocity (V_a_). In horizontal well cleanout, V_t_ and V_a_ play a significant role in suspending and transporting sands. V_t_ implies the shear force applied to solid particles in the helical flow field, which helps suspend the particle. In general, a large V_t_ will effectively prevent sands from settling down. Similarly, V_a_ implies the normal stress (pushing force) applied to solid particles. The higher V_a_ is, the faster sands move back into the flow field.

#### 3.1.1 General features of the SC-CO2 helical flow field

**Fig 7:**
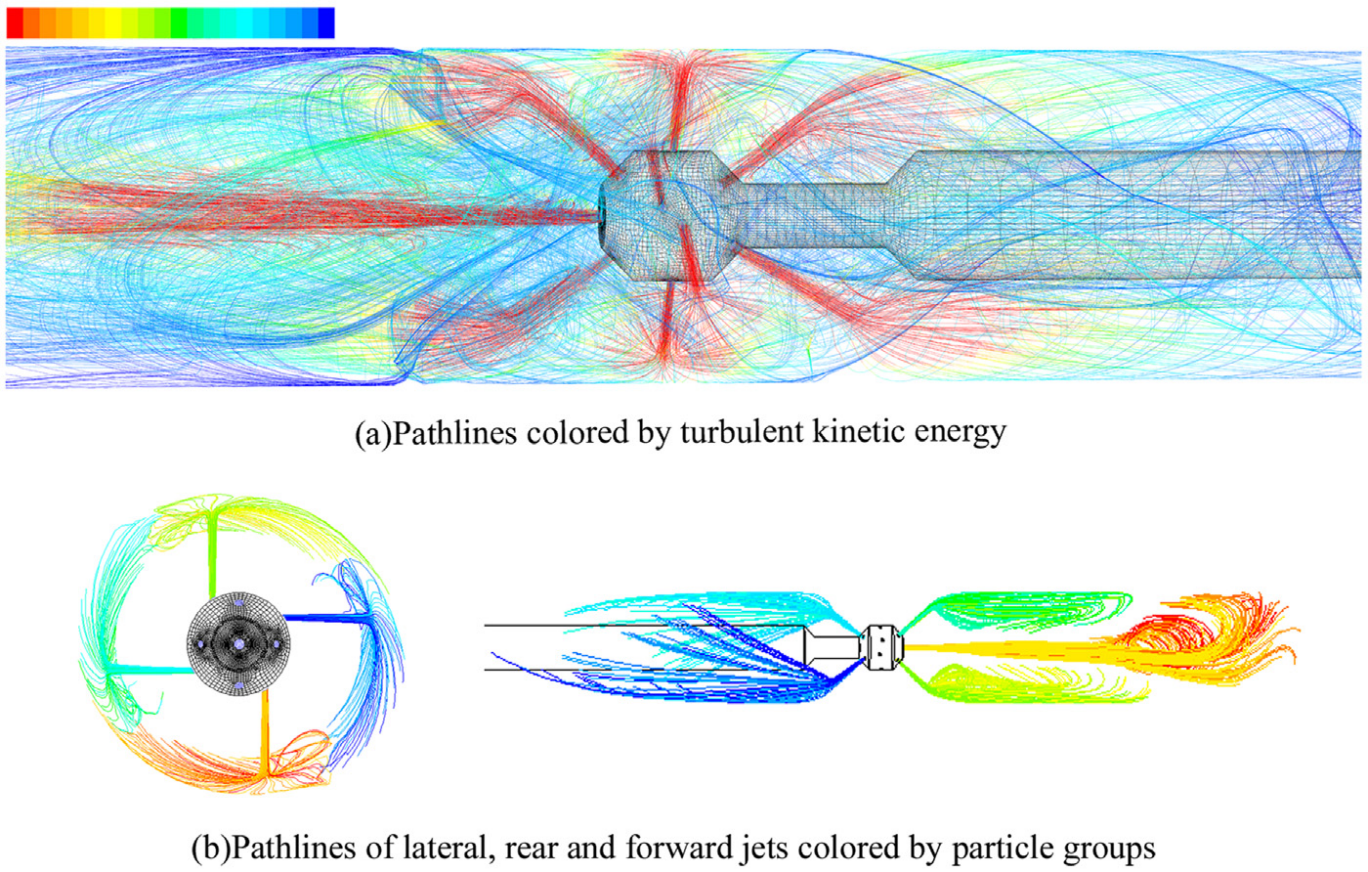
Pathlines of SC-CO_2_ helical flow field and separate jets. (A) Pathlines are colored by turbulent kinetic energy. The turbulent kinetic reaches maximum near the nozzle exits and attenuates as SC-CO_2_ flow spreads further into the annulus. The flow is from right to left at the left side of the cleaning bit, while it is left to right at the right side of the bit. (B) Pathlines are colored by particle groups. The left figure shows the process that lateral jets reach the casing wall and turn into rotational flow. The right figure displays the tracks of other three jets.

**Fig 8:**
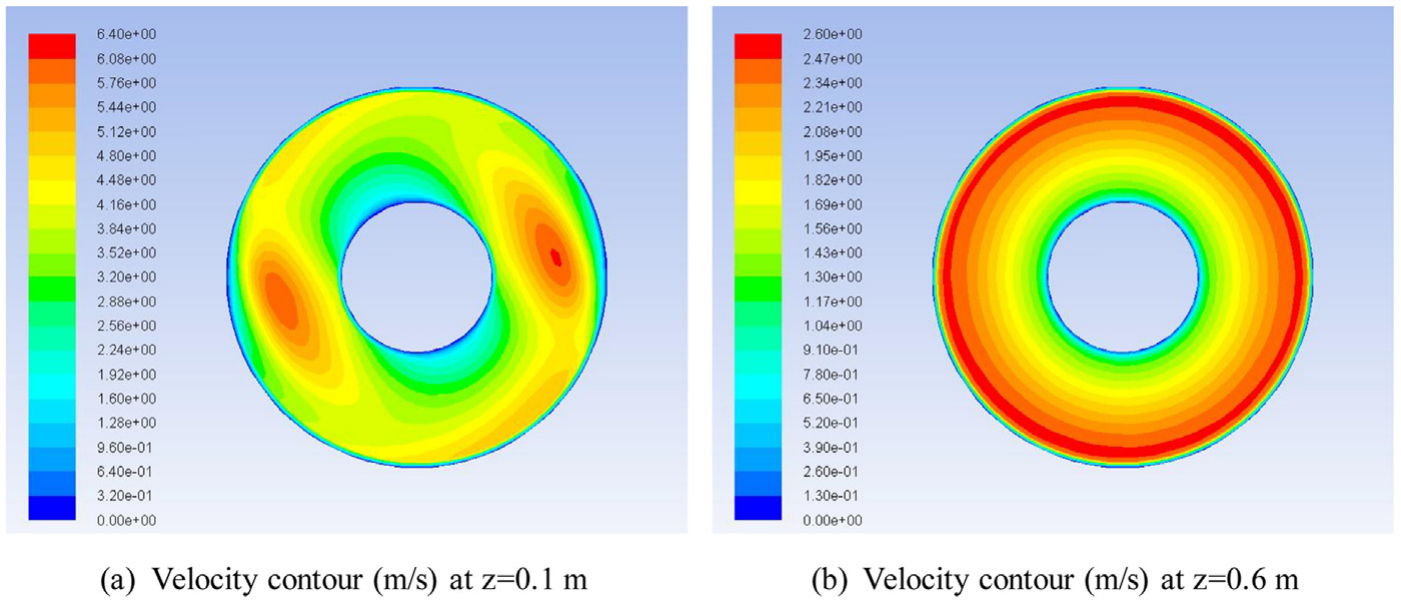
Velocity contours at different axial cross sections. (A) The flow field featured by velocity is still heterogeneous and unsteady at 0.1 m from the right side of the bit. (B) The velocity contour is annularly symmetric and thus the flow field is steady and uniformly helical.

During the cleanout operation, high-speed SC-CO_2_ jets constantly impact the wellbore, forming the annular helical flow field. Pathlines near the cleaning bit and of separate jets are displayed in Fig 6. The lateral jets impinge on the casing slantingly and generate an asymmetry overflow near the casing, which is heterogeneous and evolves to a swirling flow. Then, the initial helical flow mingles with the forward and rear jets in annulus. Based on flow pattern, the whole flow field can be divided into two zones: *transition zone* and *laminar zone*. As already mentioned, the flow field is quite chaotic and unsteady near the exit regions of the nozzles, with high turbulence (Fig 7(a)) caused by the impact of rear and forward jets on the casing and the mixing of three jets. We define the zone extending from the cleanout bit to the place where the velocity becomes annularly symmetrical as the transition zone or turbulent zone. After a certain distance from the bit, the flow field stabilizes and finally becomes uniformly helical (Fig 8(b)), which is referred as the laminar zone. Despite the different simulation parameters, we generally find that the length of the transition zone is approximately 0.6 m (Fig 8).

**Fig 9:**
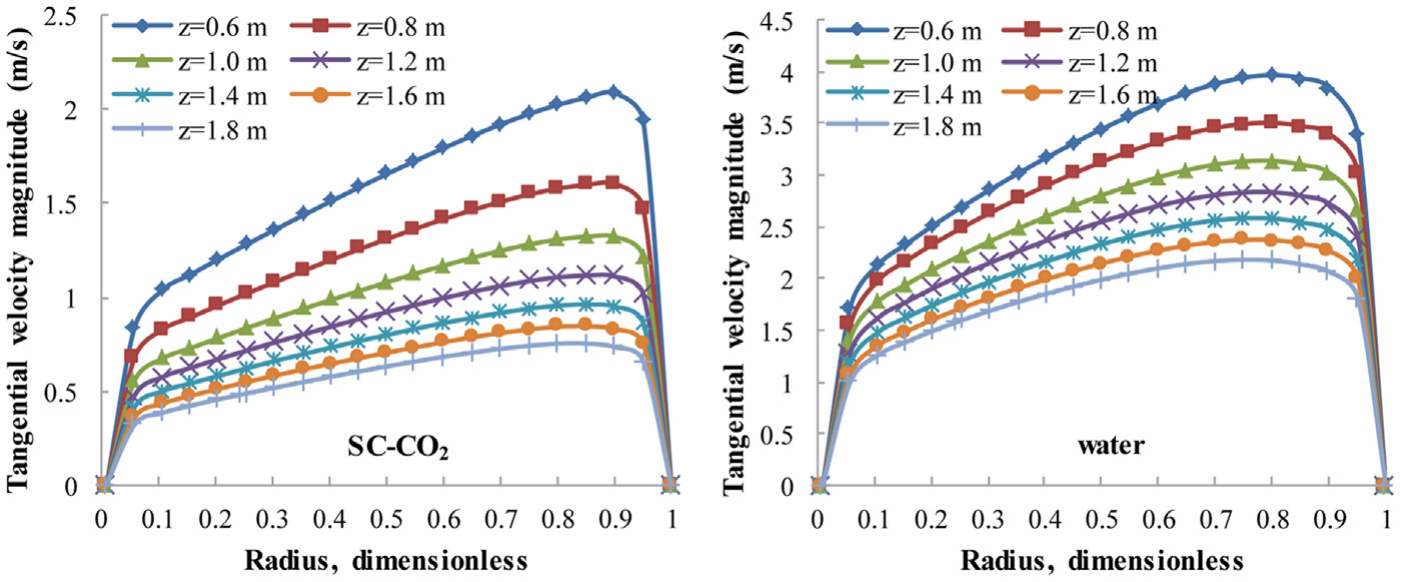
The radial distribution of V_t_ for SC-CO_2_/water flow at different cross sections. No.8 nozzle assembly; flow rate 15 L/s; ambient pressure 30 MPa; ambient temperature = jet temperature = 373 K. In cross sections, dimensionless radius is the distance from the inner wall to the measure point divided by the total length between inner and outer wall. The tangential velocity has large gradients near both sides of inner and outer walls.

Technically, the SC-CO_2_ helical flow field is more turbulent than the water helical flow field in transition zone. With the same pumping rate, temperature and ambient pressure, the velocity and density of SC-CO_2_ and water are comparable (for example, the average density of SC-CO_2_ under the condition of Fig 9 is 665 kg/m^3^, while water is about 1000 kg/m^3^). On the other hand, the viscosity of water is over one magnitude larger than that of SC-CO_2_ (the viscosity of water is 0.2838 mPa·s while that of SC-CO_2_ is only 0.0278 mPa·s under the condition of Fig 9). So from *Re = ρvd/μ* we can conclude that the SC-CO_2_ flow has higher Reynolds number (turbulence level) than water flow (In the same wellbore model, the characteristic length *d* is the same for water and SC-CO2 flow). Higher turbulence in transition zone is welcomed because it means there is more energy to stir up consolidated sands.

**Fig 10:**
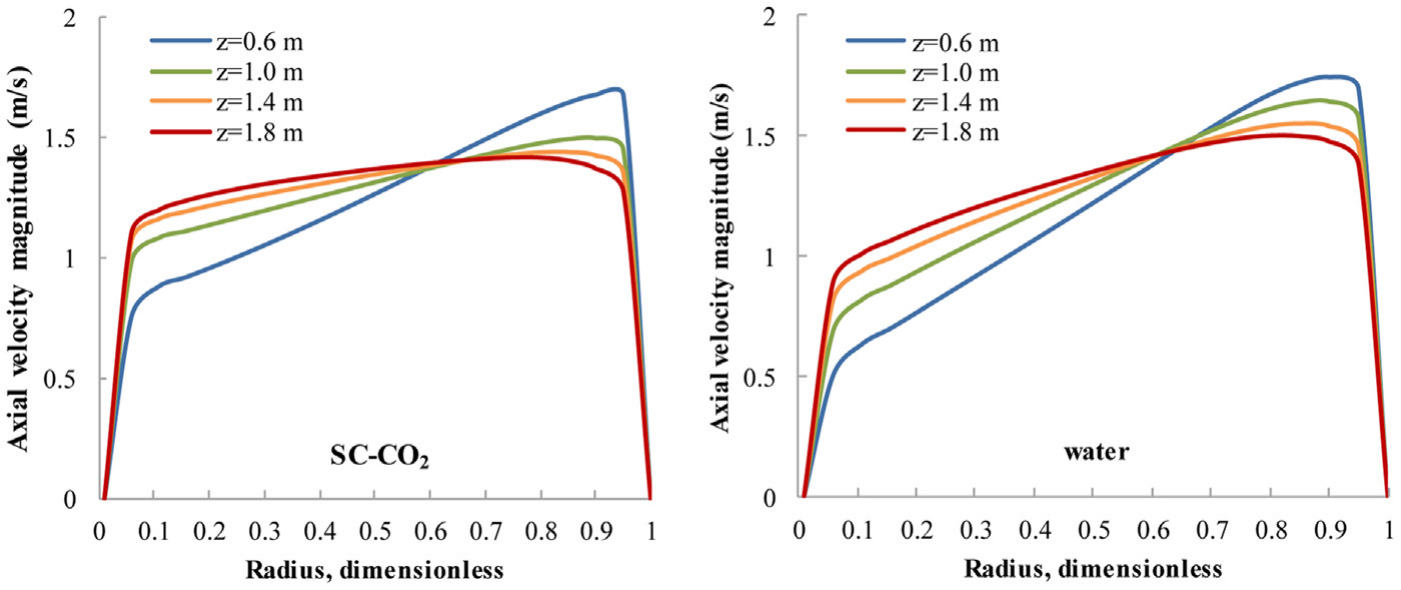
The radial distribution of V_a_ for SC-CO_2_/water flow at different cross sections. No.8 nozzle assembly; flow rate 15 L/s; ambient pressure 30 MPa; ambient temperature = jet temperature = 373 K.

In laminar zone, the low viscosity of SC-CO_2_ leads to an obvious decrease of V_t_ (Fig 9) and a more uniform distribution of V_a_ (Fig 10) in cross sections. The attenuation of V_t_ is a disadvantage in terms of suspending sands, but the uniform distribution of V_a_ means SC-CO_2_ is better at sweeping sands. The uniform V_a_ in cross sections can induce a uniform backward movement of sands, which is highly preferred in practice.

**Fig 11:**
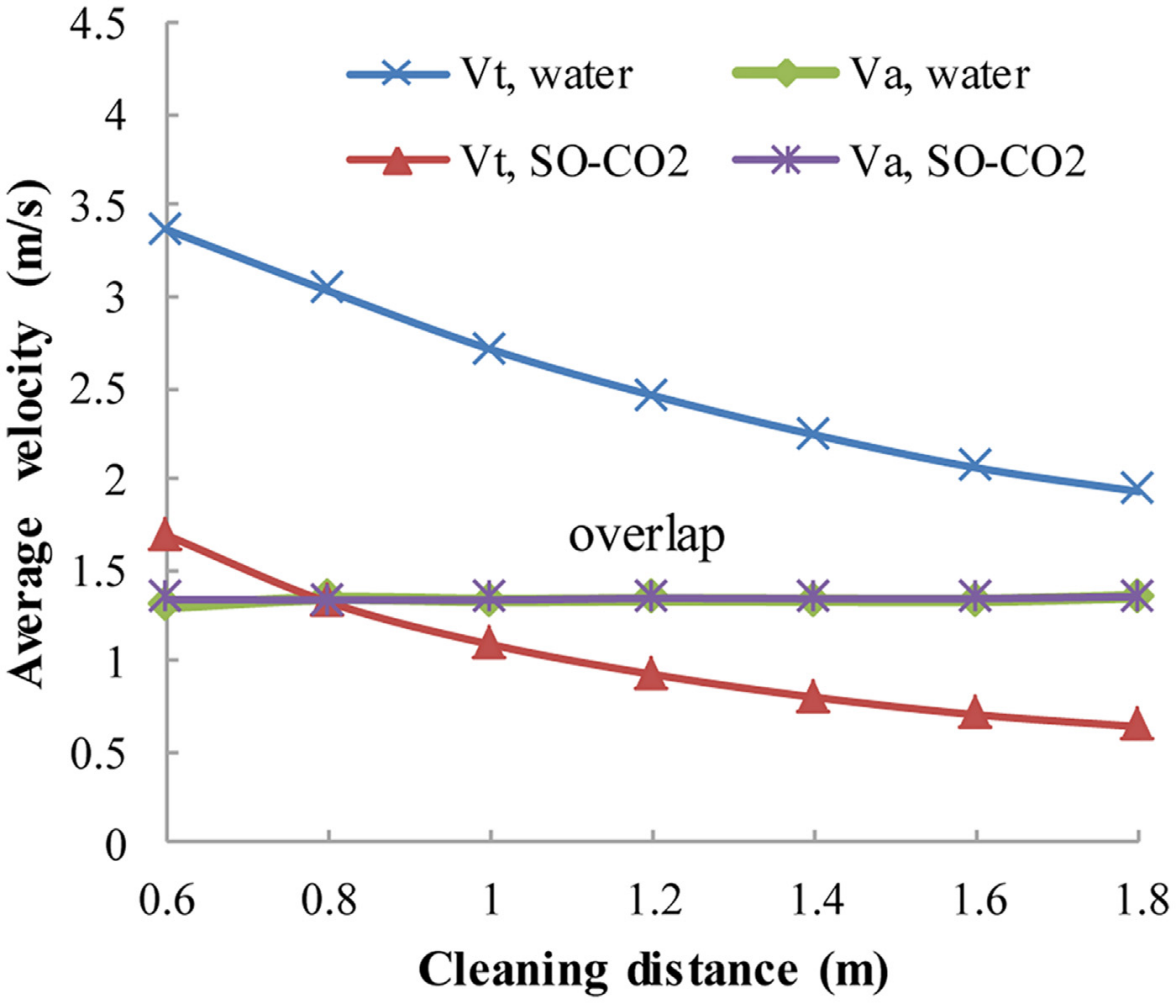
The distributions of V_a,avg_ and V_t,avg_ for SC-CO_2_/water flow along the wellbore. No.8 nozzle assembly; flow rate 15 L/s; ambient pressure 30 MPa; ambient temperature = jet temperature = 373 K.

Both V_t_ and V_a_ are close to zero near the casing and cubing, so an effective flow zone is defined as the radial ring-like region whose dimensionless radius is between 0.1 and 0.95. Then, V_t,avg_ and V_a,avg_ can be calculated as the circular-area averaged tangential/axial velocities (Fig 11) that represent V_t_ and V_a_ in different cross sections. The cleaning distance is defined as the axial distance from a certain cross section to the cleanout bit. With regard to V_a,avg_, there is little difference between the SC-CO_2_ helical flow field and the water helical flow field, whereas the V_t,avg_ of SC-CO_2_ flow is significantly smaller than that of water flow. Again, this is because SC-CO_2_ has a much smaller viscosity than water, while the densities of SC-CO_2_ and water are similar.

#### 3.1.2 Effect of different nozzle assemblies on V_a_ and V_t_

The nozzle assembly, meaning the orifice diameters of each nozzle group, affects the hydraulic power of each high pressure SC-CO_2_ jet, which consequently influences the strength of annular SC-CO_2_ helical flow.

**Fig 12:**
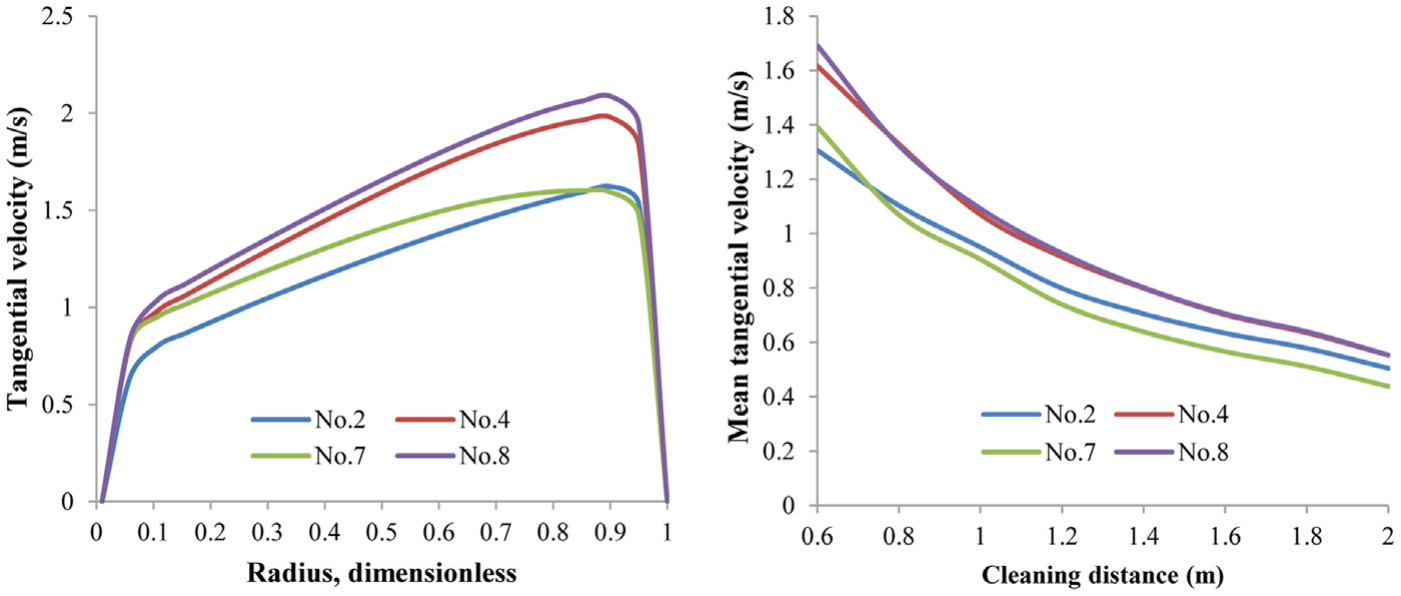
The distribution of V_t_ and V_t,avg_ with respect to different lateral nozzle sizes. Lateral nozzle diameter 3mm~5mm; flow rate 15 L/s; ambient pressure 30 MPa; ambient temperature = jet temperature = 373 K. Cleaning distance is 0.6m in the left figure.

*Lateral nozzle size*. Fig 12 exhibits the V_t_ and V_t,avg_ distributions with the same simulation parameters except lateral nozzle size. There is no monotonic relation between V_t_/V_t,avg_ and the lateral nozzle size. The No.4 nozzle assembly, with 4 mm lateral nozzles, has almost the same V_t,avg_ distribution as the No.8 nozzle assembly, whose lateral nozzles are 6 mm. Lateral jets are the main source power of rotational flow and thus determine V_t_. With larger lateral nozzles, lateral jets will have larger flow rate, which means that the flow field will be more rotational, and larger V_t_ will be achieved. Nevertheless, at the same time the nozzle equivalent diameter will be larger, leading to a reduction of lateral jets exit velocity. In this way, V_t_ is the outcome of the balance between larger lateral jets flow rate and lower exit velocity. In other words, there should be an optimal size of the lateral nozzle.

**Fig 13:**
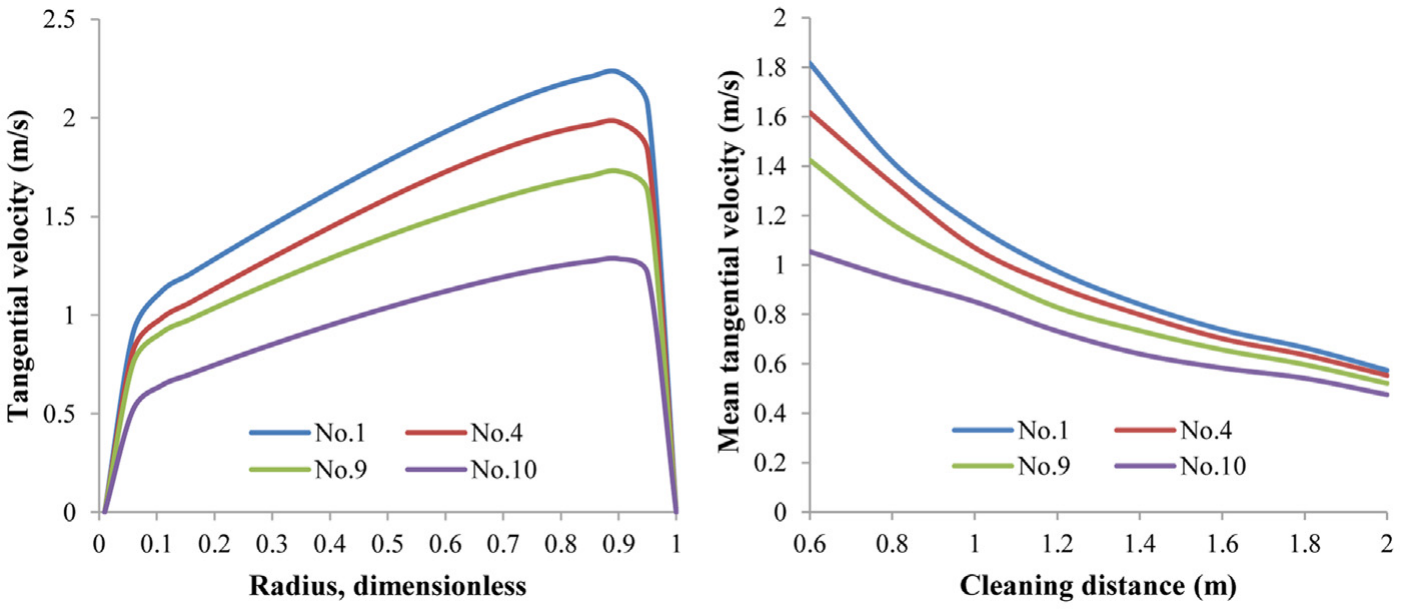
The distribution of V_t_ and V_t,avg_ with respect to different rear nozzle sizes. Rear nozzle diameter 3mm~5mm; flow rate 15 L/s; ambient pressure 30 MPa; ambient temperature = jet temperature = 373 K. Cleaning distance is 0.6m in the left figure.

*Rear nozzle size*. In contrast with the lateral nozzle, the rear nozzle size has an evident monotonic relation with V_t_. Fig 13 presents the radial distribution of V_t_ and axial distribution of V_t,avg_ varying with rear nozzle size. As the rear nozzle size increases, lateral jets have less flow rate, and V_t_ decreases significantly, reflecting the decreased helical flow strength. This decrease of V_t_ is most obvious when the rear nozzle size increases from 5 mm to 6 mm. Additionally, as the cleaning distance increases, the influence of the rear nozzle size on the helical flow strength weakens. The largest difference in V_t,avg_ occurs between the No.1 and No.10 assemblies at a cleaning distance of 0.6 m. V_t,avg_ of the No.1 assembly is 1.81 m/s, whereas that of the No.10 assembly is 1.05 m/s. This can lead to a distinct difference in cleanout efficiency. Minimizing the rear nozzle size seems a possible way to improve the suspending ability of the helical flow field. However, if this is the case, the sweeping efficiency will be damped as the distributed flow rate for rear nozzles decreases. Actually, the rear jets can weaken V_t_ but enhance V_a_ in the annular helical flow.

**Fig 14:**
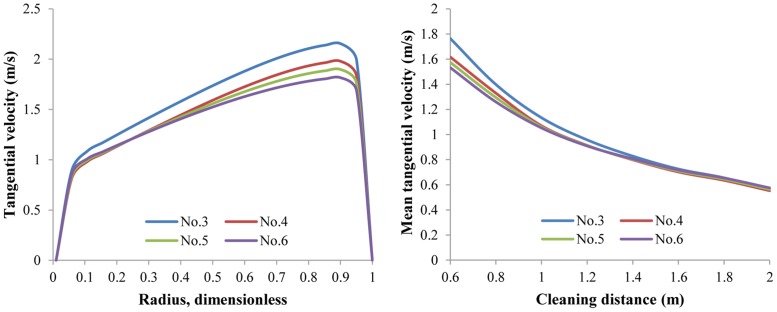
The distribution of V_t_ and V_t,avg_ with respect to different forward nozzle sizes. Forward nozzle diameter 3mm~5mm; flow rate 15 L/s; ambient pressure 30 MPa; ambient temperature = jet temperature = 373 K. Cleaning distance is 0.6m in the left figure.

*Forward nozzle size*. Compared to the rear nozzle, the forward nozzle size has less influence on V_t_ distributions (Fig 14). As forward nozzle size increases, V_t,avg_ has a much lower tendency to decrease. Likely, minimizing forward nozzle size for the purpose of larger V_t_ is not the best way because the forward jets require enough flow rate and energy to break up solid sediments in the front of the cleaning bit.

In conclusion, reducing the rear/forward nozzle size or increasing the lateral nozzle size can enhance the strength of annular helical flow by increasing V_t_. However, the jets for penetration and sweeping will be reduced. Thus, there exists an optimal nozzle assembly that can provide the best combination of V_t_ and V_a_. This issue will be addressed later in the BP-ANN optimization model.

#### 3.1.3 Effect of flow rate on V_a_ and V_t_

**Fig 15:**
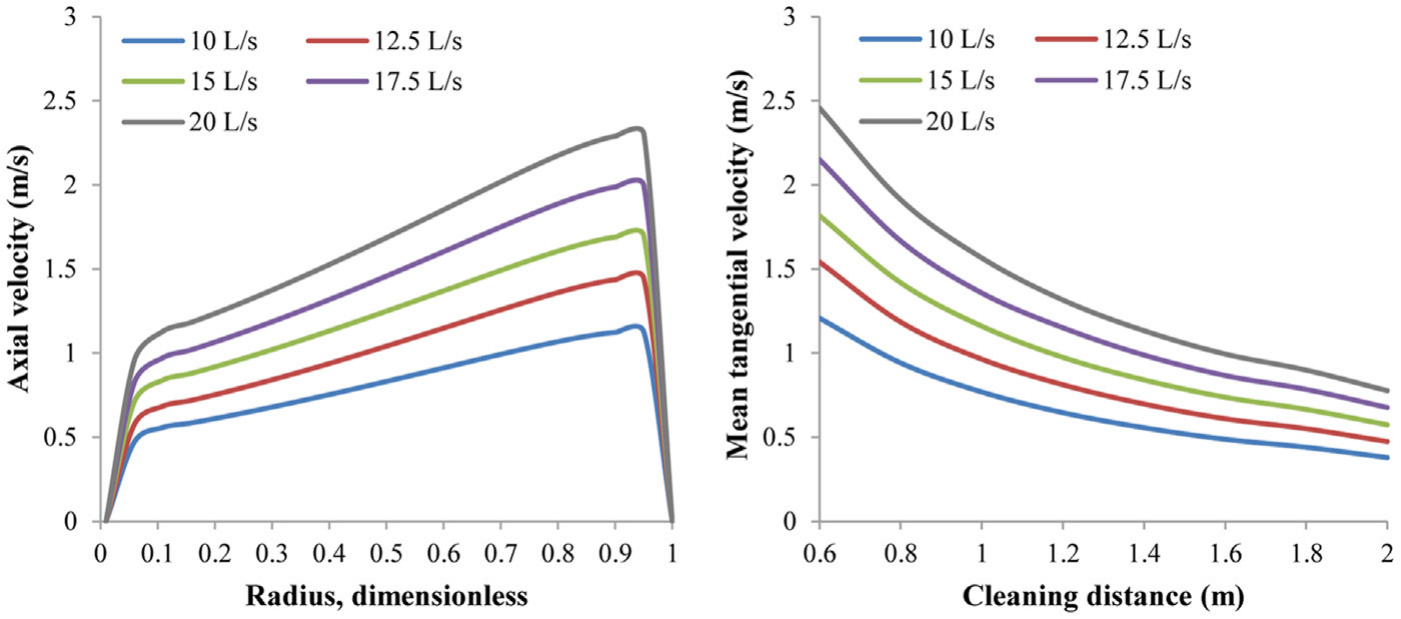
The distribution of V_a_ and V_t,avg_ with respect to different flow rates. Nozzle assembly No.1; flow rate 10 L/s~20 L/s; ambient pressure 30 MPa; ambient temperature = jet temperature = 373 K.

Naturally, a higher flow rate will induce larger V_t_ and V_a_. We find that during the simulation, both V_t_ and V_a_ are almost directly proportional to the flow rate, as are V_t,avg_ and V_a,avg_ (Fig 15). In this sense it seems that the flow rate should be as high as possible. However, a high flow rate requires greater pump and increases energy costs. To strike an economical balance between velocities and flow rate cost, BP-ANN optimization model can be applied; this will be discussed in section 3.2.

#### 3.1.4 Effect of ambient pressure on V_a_ and V_t_

**Fig 16:**
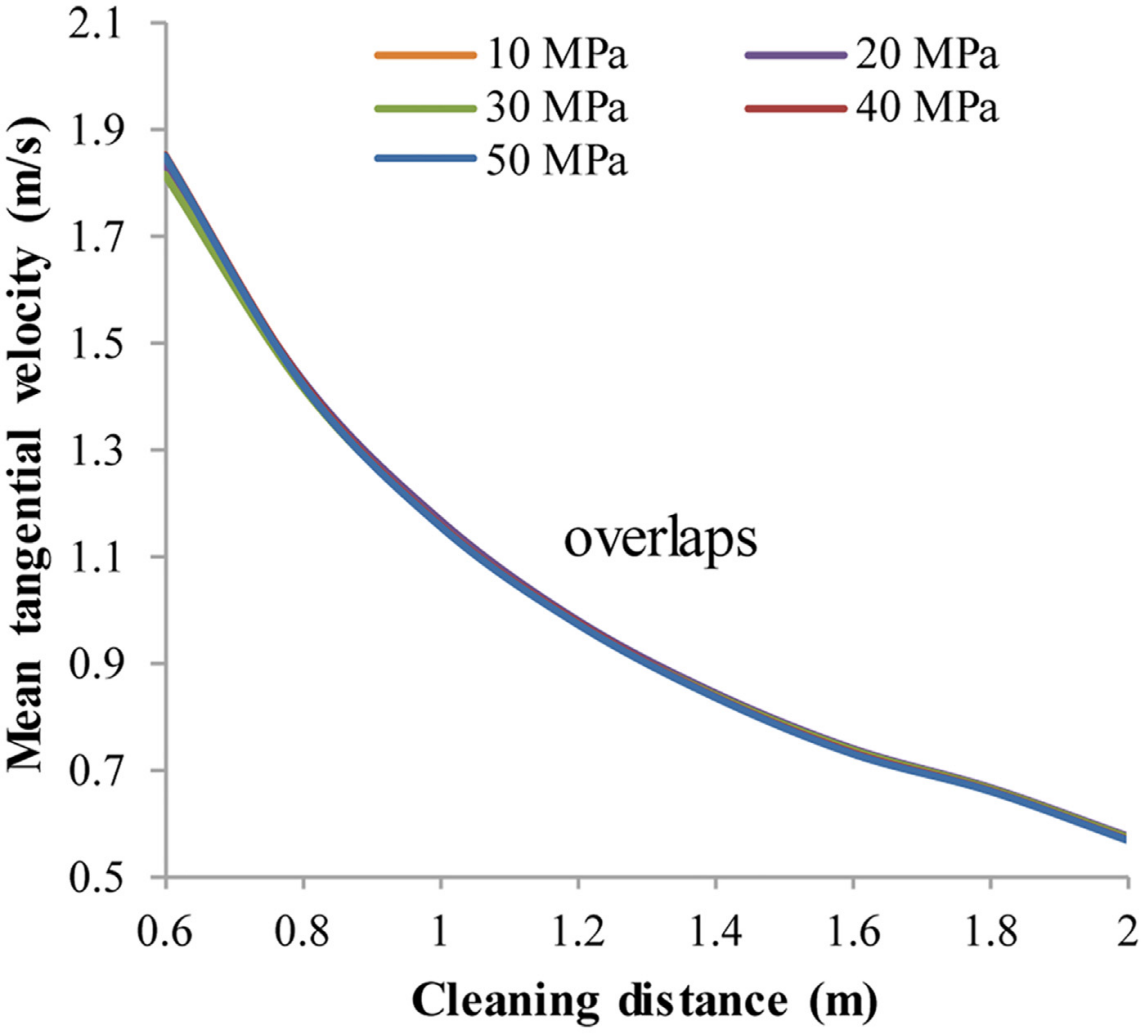
The distribution of V_t,avg_ with respect to different ambient pressures. No.1 nozzle assembly; flow rate 15 L/s; ambient pressure 10 MPa~30 MPa; ambient temperature = jet temperature = 373 K.

Ambient pressure is found to be the least influential factor. In fact, V_t,avg_ and V_a,avg_ hardly change with different ambient pressures (Fig 16). Considering the relationship between ambient pressure and TVD, this means that the SC-CO_2_ helical flow cleanout can be performed in horizontal wells whose vertical depth is no more than 5000 m, assuming that the pressure gradient is 1 MPa/100 m.

#### 3.1.5 Effect of jet and ambient temperature on V_a_ and V_t_

Temperature is a minor factor in water helical flow cleanout because the properties of water hardly change with temperature. In contrast, the physical properties of SC-CO_2_ are sensitive to temperature. Ambient temperature is defined as the temperature of the formation, casing and tubing, and jet temperature is the temperature of jets when they squirt from the nozzles.

**Fig 17:**
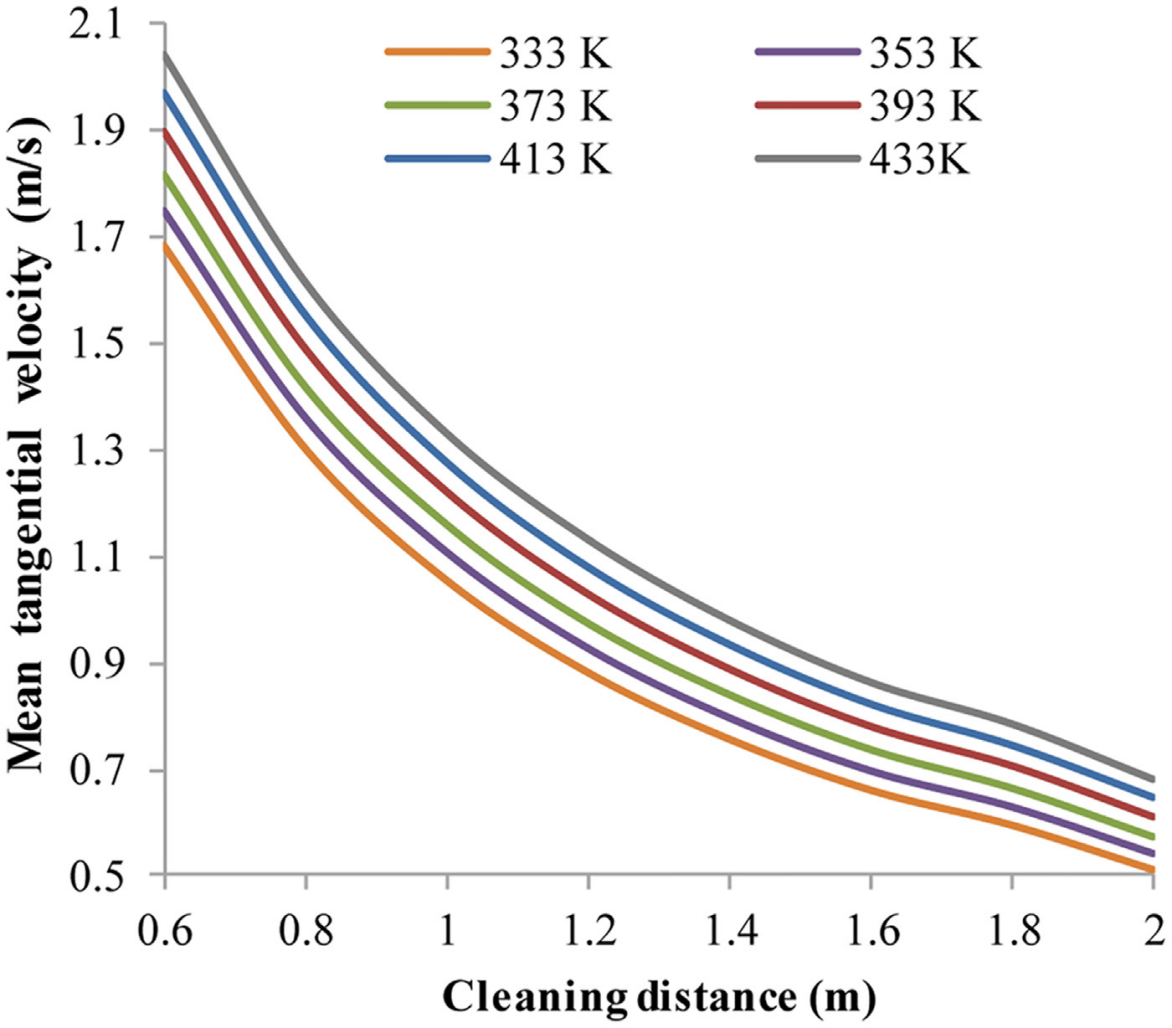
The distribution of V_t,avg_ with respect to different ambient temperatures. No.1 nozzle assembly; flow rate 15 L/s; ambient pressure 30 MPa; jet temperature 373 K; ambient temperature 333 K~433 K.

Ambient temperature has an approximately linear relationship with V_t,avg_ (Fig 17) and V_a,avg_ at a certain cleaning distance. V_t,avg_ increases with higher ambient temperature. At cleaning distances of 0.6 m and 2.0 m, the percentages of V_t,avg_ with ambient temperature increasing from 333 K to 433 K, increase by 16.8% and 25.7%. Furthermore, the percentages of V_a,avg_ increase by 26.9% and 32.0%. The increased ambient temperature has a greater influence on V_a,avg_ than V_t,avg_. Moreover, at larger cleaning distances, the influence of ambient temperature is enhanced.

**Fig 18:**
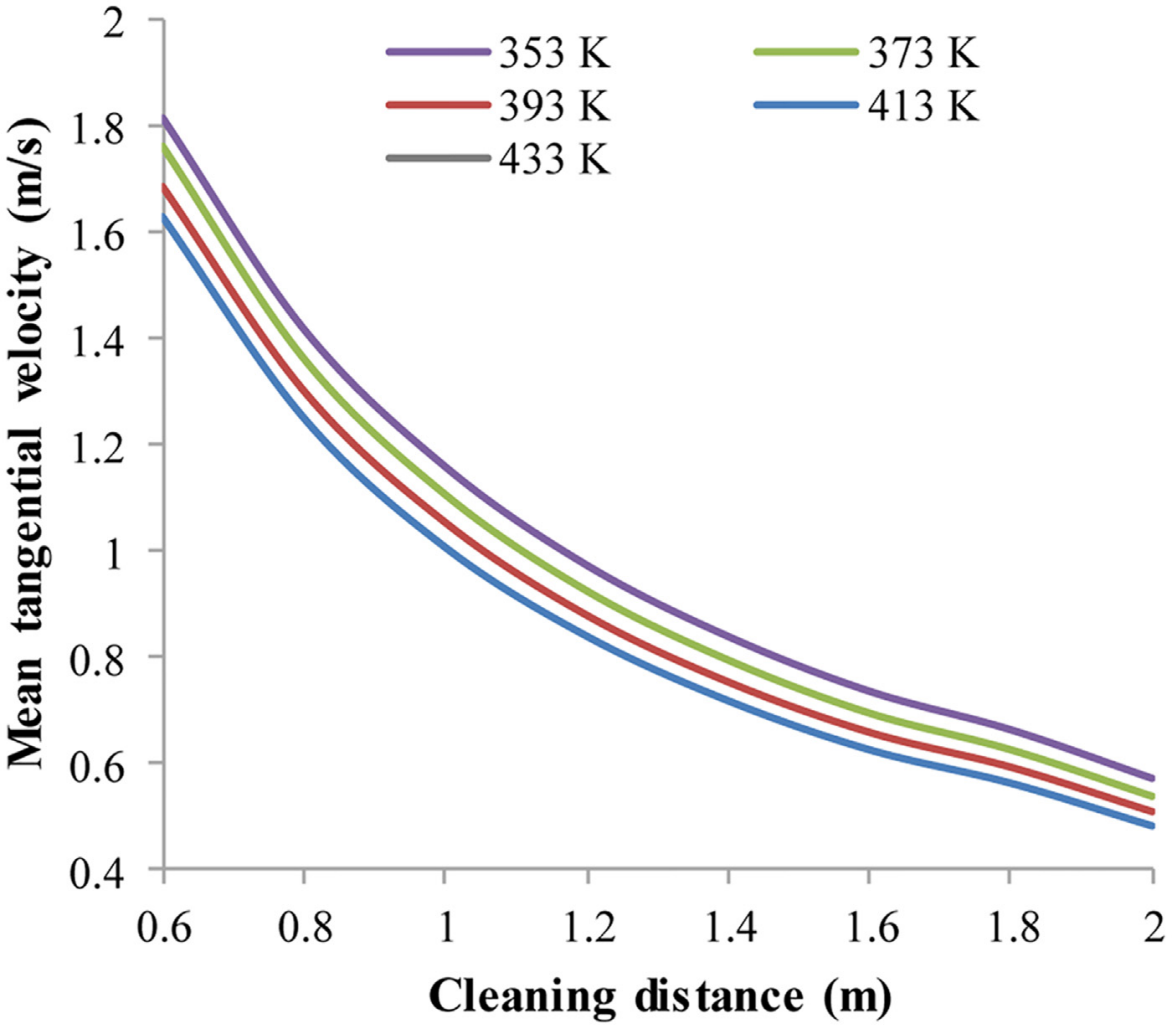
The distribution of V_t,avg_ with respect to different jet temperatures. No.1 nozzle assembly; flow rate 15 L/s; ambient pressure 30 MPa; ambient temperature 373 K; jet temperature 353 K~433 K.

Analogously, V_t,avg_ and V_a,avg_ also have proportional relationships with jet temperature (Fig 18). However, the difference is that V_a,avg_ and V_t,avg_ decrease with increasing jet temperature. At a cleaning distance of 0.6 m, with the jet temperature increasing from 353 K to 433 K, the percentages of V_t,avg_ and V_a,avg_ decrease by 13.6% and 20.1%. At a cleaning distance 2.0 m, the percentages of V_t,avg_ and V_a,avg_ decrease by 21.1% and 24.1%. Again, V_a,avg_ is more sensitive to jet temperature than V_t,avg_, and the velocities are influenced more by jet temperature at larger cleaning distances.

**Fig 19:**
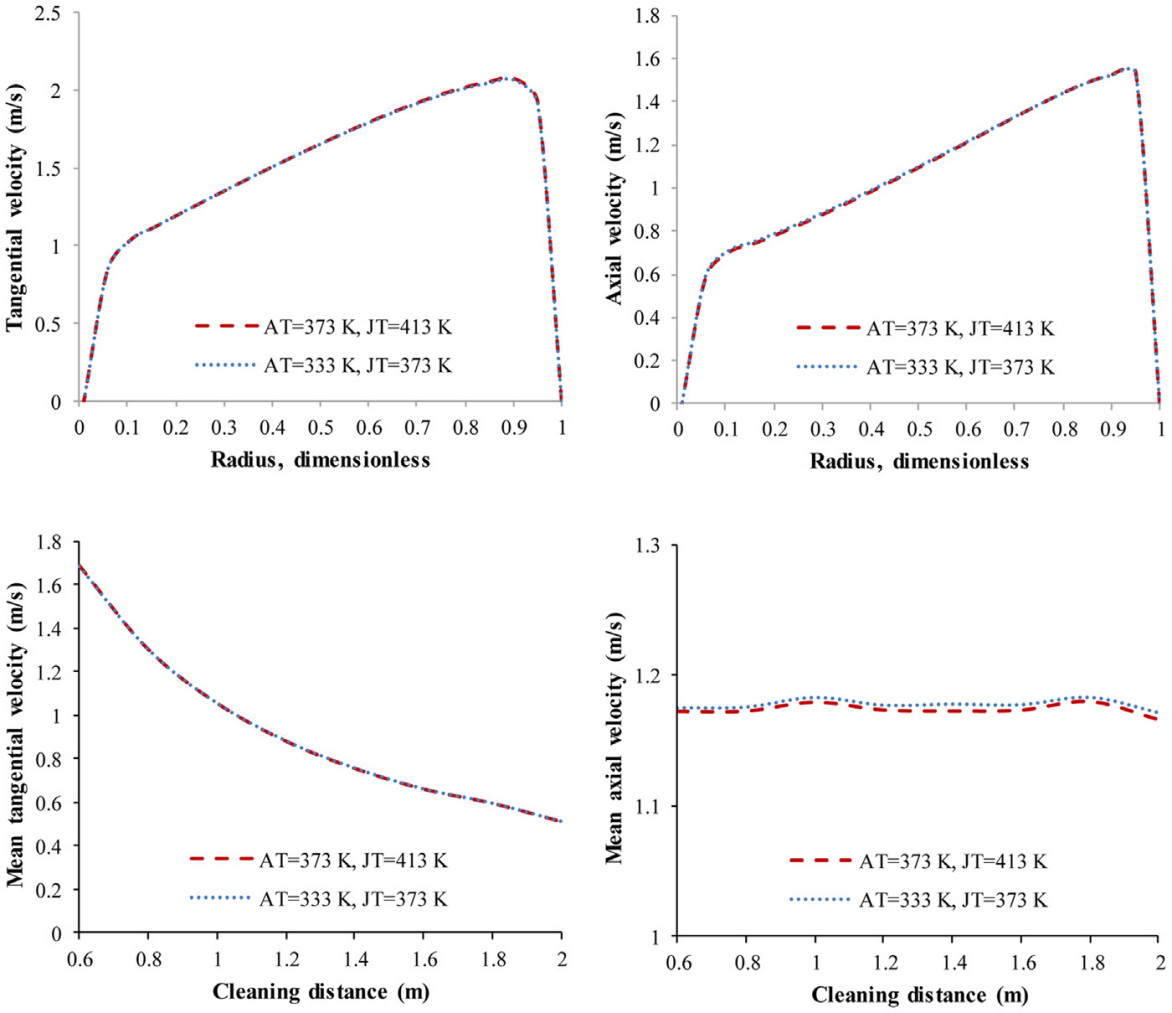
V_t_, V_a_, V_t,avg_ and V_a,avg_ with the same ambient/jet temperature difference. No.1 nozzle assembly; flow rate 15 L/s; ambient pressure 30 MPa; AT is ambient temperature; JT is jet temperature.

Actually, ambient temperature and jet temperature share more common properties. The change of ambient/jet temperature is, substantially, no more than the change in the difference between these two temperatures. We picked cases that had the same difference between the ambient and jet temperatures and then compared the distributions of V_t_ and V_a_. In the two simulation cases where the ambient/jet temperatures are 373 K/413 K and 333 K/373 K—i.e., the difference between the ambient and jet temperatures is -40K—the distributions of V_a_, V_t_, V_t,avg_ and V_a,avg_ are nearly identical (Fig 19). The same phenomenon occurs in all other cases with the same temperature difference. We can conclude that V_t_ and V_a_ depend on the difference between the ambient and jet temperatures, not on these two variables independently.

In SC-CO_2_ drilling, liquid CO_2_ is pumped into the drilling string. It is heated and pressurized as CO_2_ goes deep underground. When the critical temperature and pressure are reached, CO_2_ achieves a supercritical state. The relationship between the surface temperature of liquid CO_2_ and the temperature of SC-CO_2_ jets can be clarified by wellbore heat transfer models [60,61]. Thus, it is feasible to modify the surface temperature based on the design of the jet temperature.

In this section, the features of SC-CO_2_ helical flow are investigated and compared with water helical flow. SC-CO_2_ helical flow is feasible in specific cleanout cases such as water-sensitive and low-permeability formations. In terms of nozzle assembly, there is an optimal size for the lateral nozzle to produce the best V_t_. Though a smaller rear/forward nozzle size is beneficial to improve V_t_, it may not guarantee the ultimate performance of cleanout. Better flow rate is able to boost V_t_ and V_a_ but requires extra pump and energy costs. Ambient pressure has little influence on SC-CO_2_ helical flow. V_t,avg_ and V_a,avg_ improve with higher ambient temperature or lower jet temperature. V_t_ and V_a_ depend on the difference between the ambient and jet temperatures, not on these two variables independently.

### 3.2 Performance of the BP-ANN model

#### 3.2.1 BP-ANN data fitting

**Fig 20:**
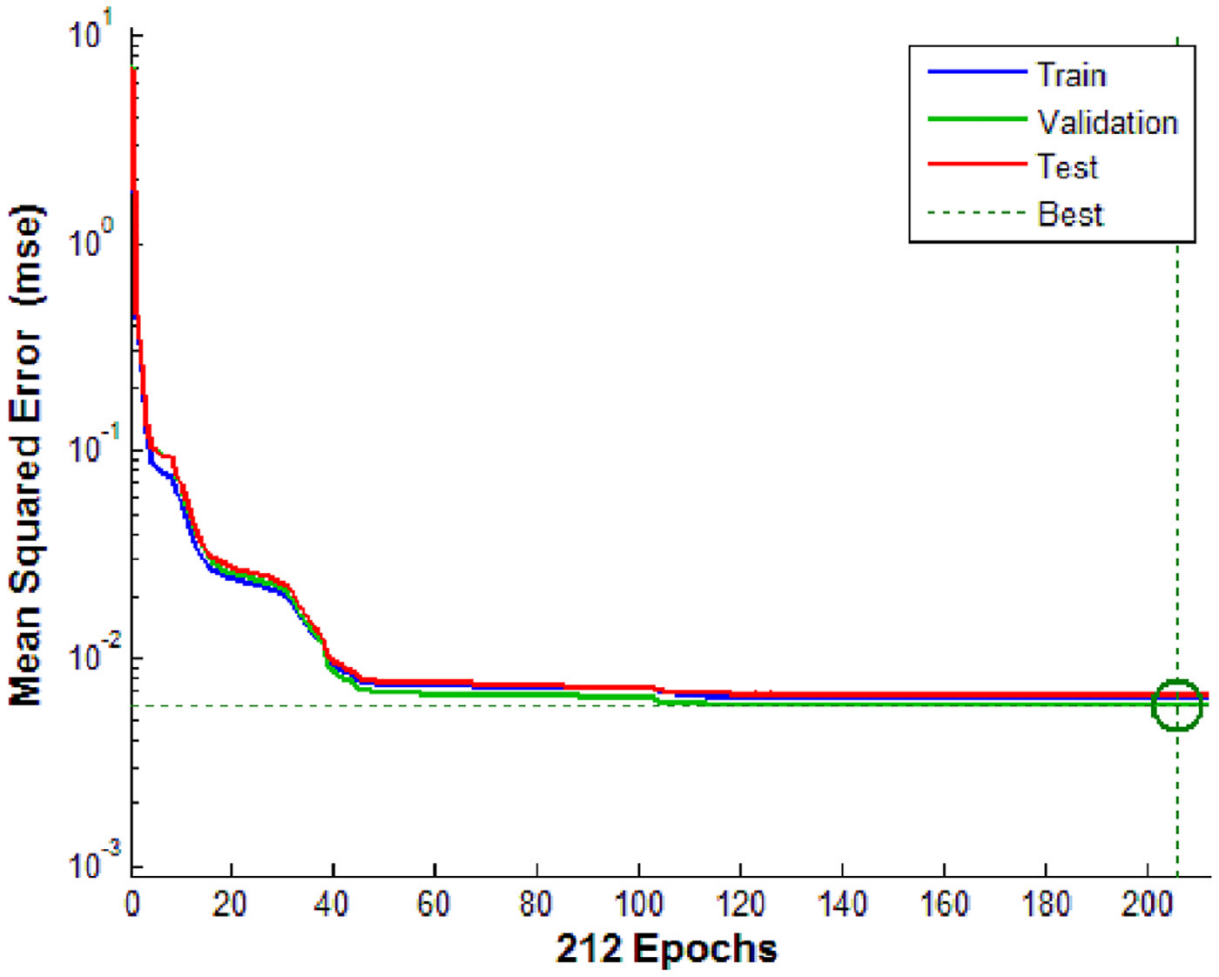
MSE vs. iteration number. The plots of train, validation and test process are quite close, indicating good performance of ANN. The smallest MSE during validation is 0.0059 at epoch 206. Validation stops when MSE keeps invariable for some epochs.

**Fig 21:**
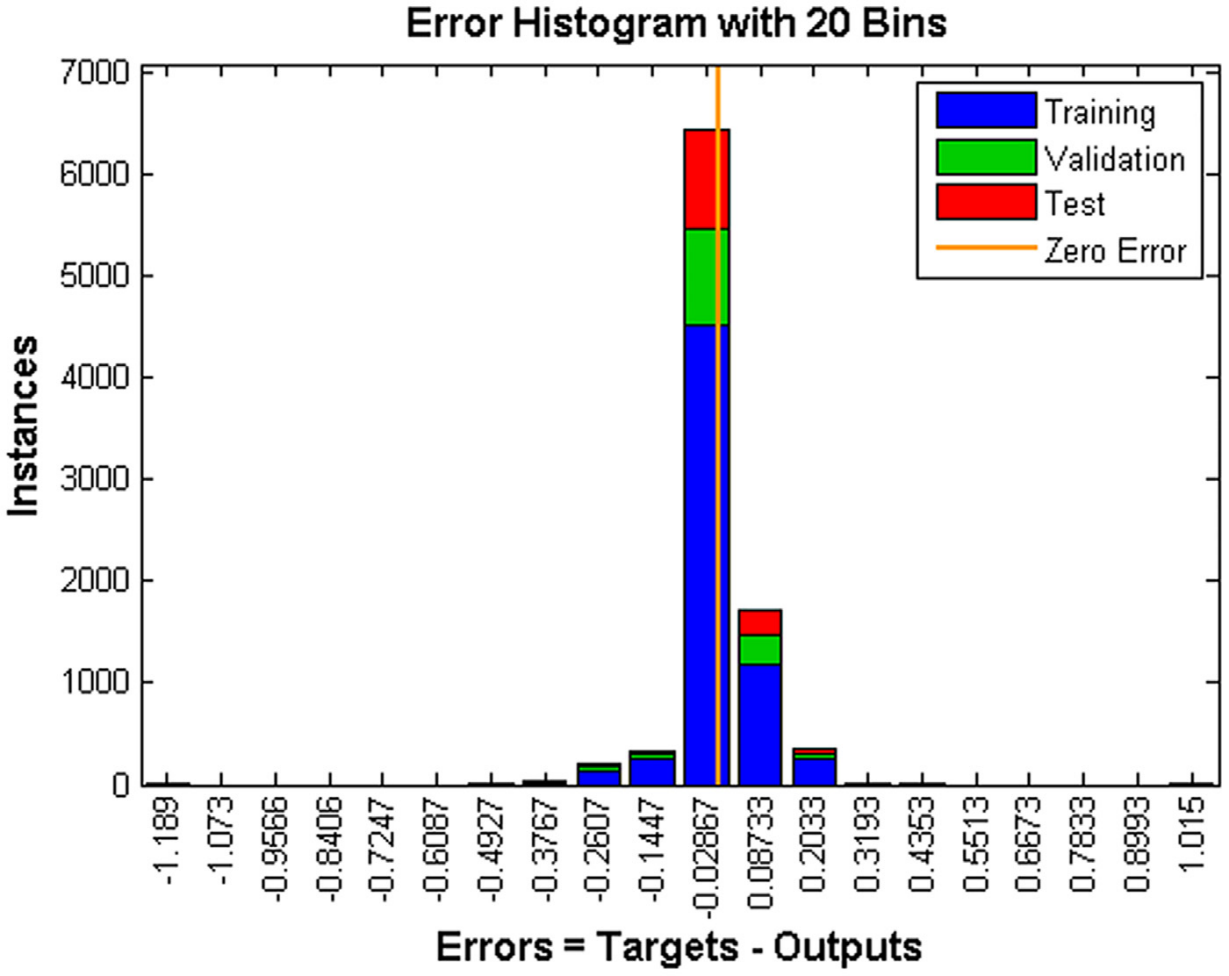
Error histogram.

**Fig 22:**
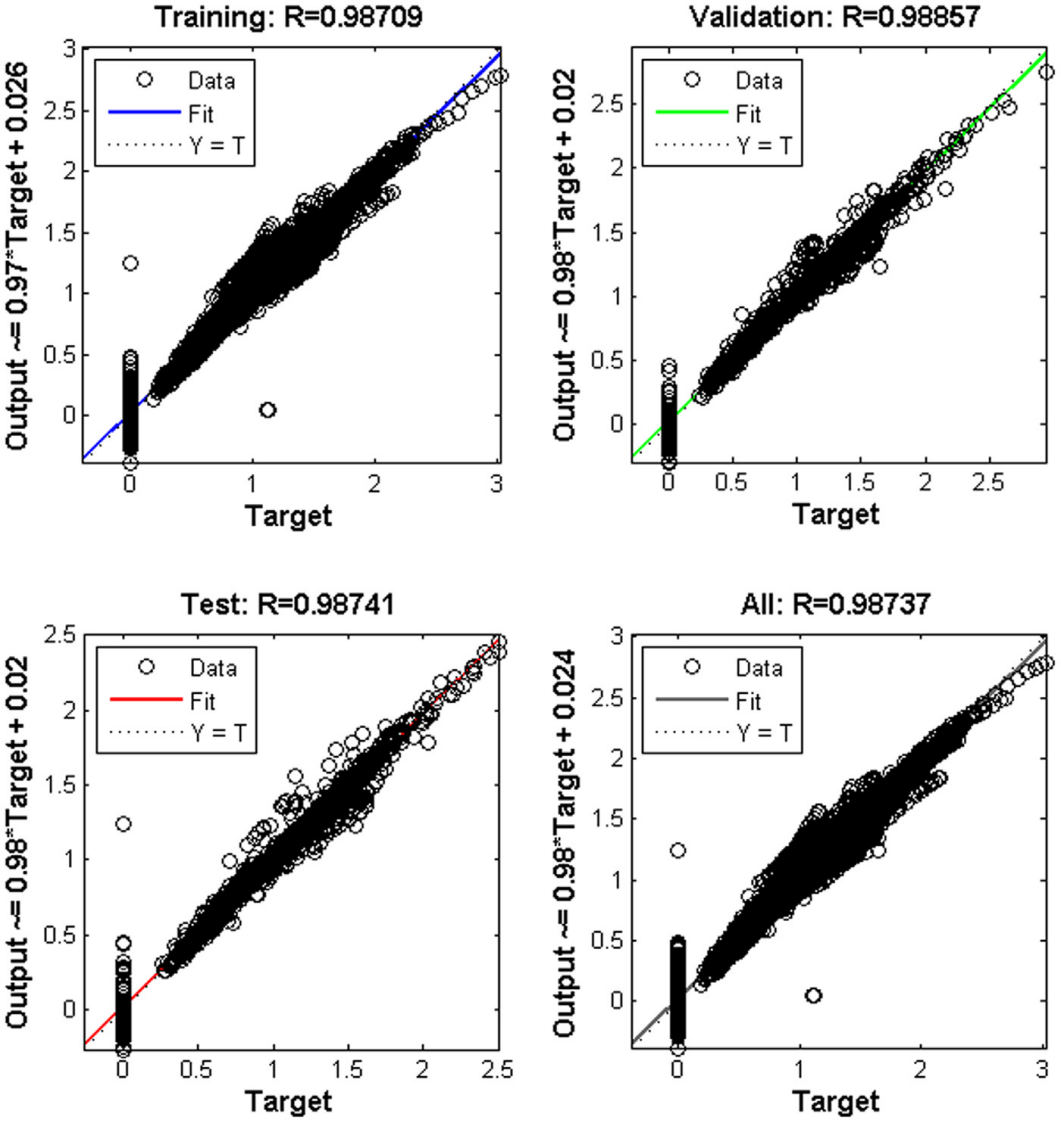
Regression plots. R is coefficient of determination. Target is the true velocities of the simulated flow field while output is the results of the ANN model that should approach target. Points far away from the diagonal are ‘noise’ points.

In total, 4563 samples covering 28 cases were inputted to the BP-ANN. The training, test and validation processes were performed successfully with sufficient accuracy. There were 212 iterations in all, with a decent rate of convergence, and the best validation performance of MSE was 0.0059 (Fig 20). The error histogram (Fig 21) shows that most absolute errors are within a small range near zero, which is a sign of good performance for the BP-ANN. Moreover, the R^2^ values of the training, test, validation and combination thereof are quite close to 1 (Fig 22), indicating perfect regression between the outputs and targets. It should be noted that in regression plots, there are several points that are far away from the diagonal (regression line). These points are abovementioned ‘noise’ points resulting from either improper sampling position or perturbance of the helical flow field. In this model, these ‘noise data’ are not included, thus avoiding the over-fit problem.

#### 3.2.2 Comparison between SVM and ANN

SVR (Support Vector Regression) based on SVM method was applied to fit the operation parameters with V_t_ and V_a_. RBF (Radial Basis Function) was chosen as the kernel function. Since one E-SVR can only have one output, two independent E-SVRs were trained to match V_t_ and V_a_, respectively. Similarly as training BP-ANN, total data was divided into two parts: 70% was used for training while 30% was used for validation.

BP-ANN and E-SVR were compared in terms of time consumption and accuracy. The training time for two E-SVRs combined was 8.5s while that for BP-ANN was 23s. Although it seemed that E-SVRs required less time to train, it was finding the best values of *g* (gamma) for RBF kernel function and *c* for loss function that costed massive time. Two methods were used to find the best g and c based on cross validation: Grid Method (GM) which consumed nearly an hour; PSO (Particle Swarm Optimization) method which consumed more than 16 hours in total. The sets of ‘best’ *g* and *c* were found to be 256 and 16 for both V_t_ and V_a_ by GM. By PSO, best *g*s were found to be 100 for V_t_ and V_a_ while *c*s were 31.8885 for V_t_ and 36.0296 for V_a_. E-SVR trained with PSO parameters had better accuracy (total R^2^ was 0.9530 for PSO-trained E-SVR while that was 0.9439 for GM-trained E-SVR). So E-SVR with parameters by PSO seemed to be the best SVR model for this problem, which had R^2^ of 0.9480 and 0.9580 for training and validation. The best MSE of E-SVR was 0.1039 while that of BP-ANN was 0.0059. The R^2^ for BP-ANN training, validation and test were 0.9871, 0.9886 and 0.9874 with total R^2^ equal to 0.9874. Thus it can be concluded BP-ANN had better performance for this problem than SVM method, both in terms of time (23s vs 16 hours plus) and accuracy (0.9530 vs 0.9874 of R^2^). And that’s why we used BP-ANN instead of SVM.

#### 3.2.3 Optimization strategies

Once the best match of BP-ANN model is achieved, it can produce predicted V_t_ and V_a_ based on input operation parameters. Combined with corresponding requirements and strategies, optimization of operation parameters can be achieved. Thus, the trained BP-ANN model embedded with strategies is called *BP-ANN optimization model*. Following are two important strategies for the BP-ANN optimization model.

**Fig 23:**
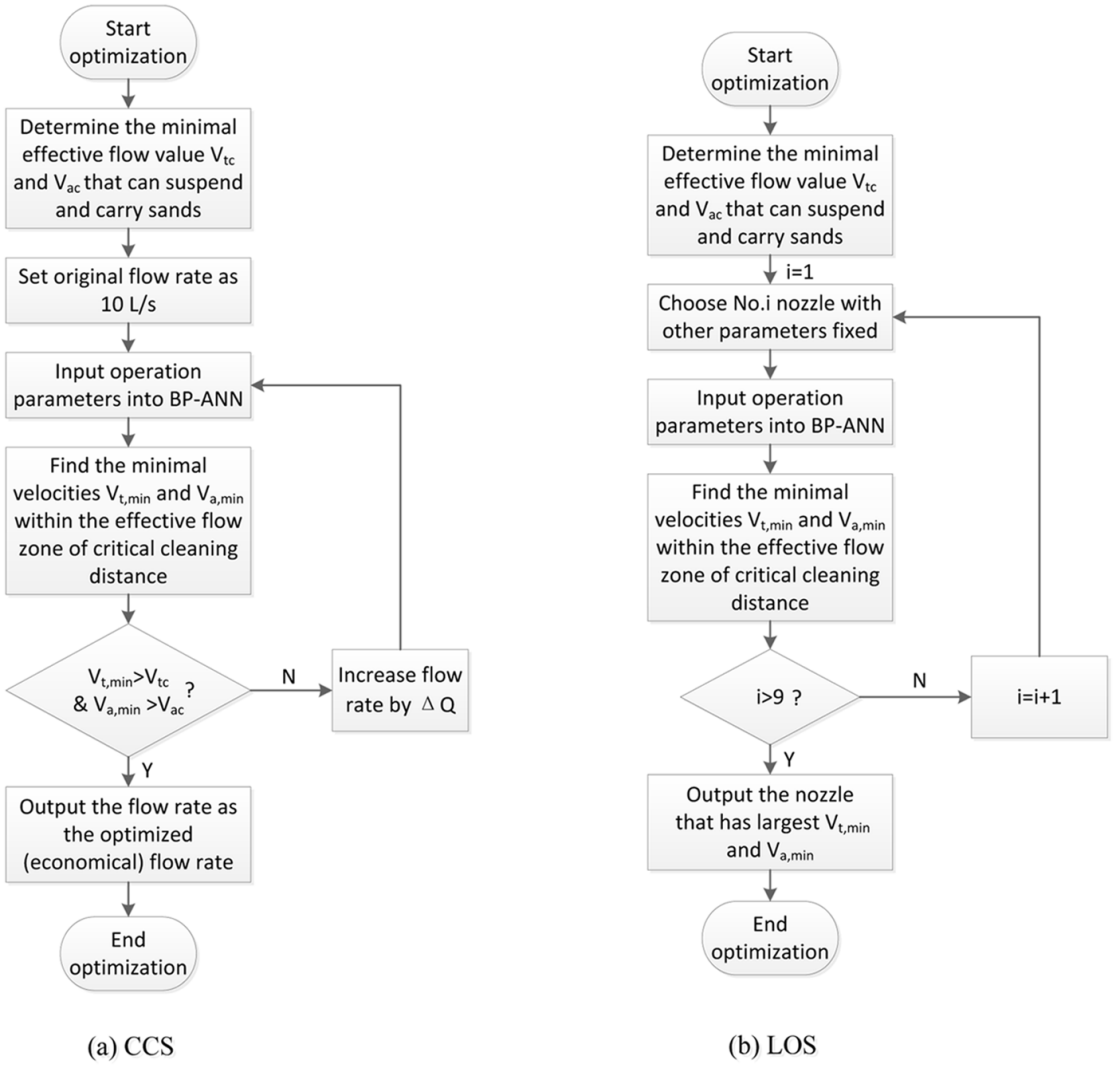
Shematic diagram of BP-ANN optimization strategies.

*Cost-Saving Strategy (CSS)*. Flow rate is one of the main factors that determine the cost of cleanout. Maintaining a large flow rate requires heavy-duty pumps and excessive energy, which are often expensive. To fulfill the cleanout task, it is only necessary to make sure that the intensity of flow field within a critical cleaning distance is big enough to carry sands. In this way, the essence of CSS is to find the minimal flow rate (pumping rate) that keeps V_t,min_ and V_a,min_ (the minimum tangential and axial velocity) within the effective flow zone of critical cleaning distance above critical values (V_tc_ and V_ac_, below which sands cannot be carried sufficiently), so that sands can be suspended and carried by the helical flow field. The critical values for cleaning distance, V_t_ and V_a_ are functions of wellbore geometry, flushing fluid property and sand size, which is beyond the scope of present study. V_ac_ sets the upper boundary of the cleanout time and should be decided according to the design requirement. After determining V_tc_ and V_ac_, we can increase the input flow rate from 10 L/s gradually and get the output increasing V_t,min_ and V_a,min_ from BP-ANN model with other input parameters fixed. The minimal flow rate required is obtained when V_t,min_ and V_a,min_ reach V_tc_ and V_ac_. The process of parameters optimization using CSS is illustrated in Fig 23(a).

*Local Optimal Strategy (LOS)*. Practically, it is conceivable that only limited types of tools and equipment are available in well sites. In this situation, although the optimal operation parameter set that can have the largest V_t_ and V_a_ cannot be obtained, it is still beneficial if a local optimal set of parameters can be decided. One example is to determine the optimal nozzle assembly when there are only limited types of pumps and cooling equipment available, that is, when the flow rate and jet temperature have limited options. On the one hand, there is the requirement that V_tc_ and V_a,c_ should be satisfied in the first place; on the other hand, a stronger helical flow field will definitely result in better cleanout performance. In other words, the aims of LOS are: 1) find nozzle assemblies that can meet the requirement of V_tc_ and V_ac_; 2) find the best nozzle assembly by which V_t,avg_ and V_a,avg_ in the effective zone can be maximized. The process of parameters optimization using CSS is illustrated in Fig 23(b).

#### 3.2.4 Optimization cases

*Case 1*. *CSS*. Assume that there is a horizontal well that requires SC-CO_2_ helical flow cleanout. The TVD of the well is 3500 m; the geothermal gradient is 2°C/100 m; the surface temperature is 20°C; the pressure gradient is 1 MPa/100 m; the No.3 nozzle assembly is used; the jet temperature is 373 K; the critical cleaning distance is 2.5 m; and V_tc_ and V_ac_ are 0.3 m/s and 0.9 m/s, respectively. Recall the BP-ANN model; it is easy to find the minimum flow rate that meets the requirement of the critical velocities is 15.4 L/s. The corresponding minimum pump rate can thus be selected after other hydraulic losses are considered. In the end, the cleanout can be performed economically with the lowest pump and energy cost.

**Fig 24:**
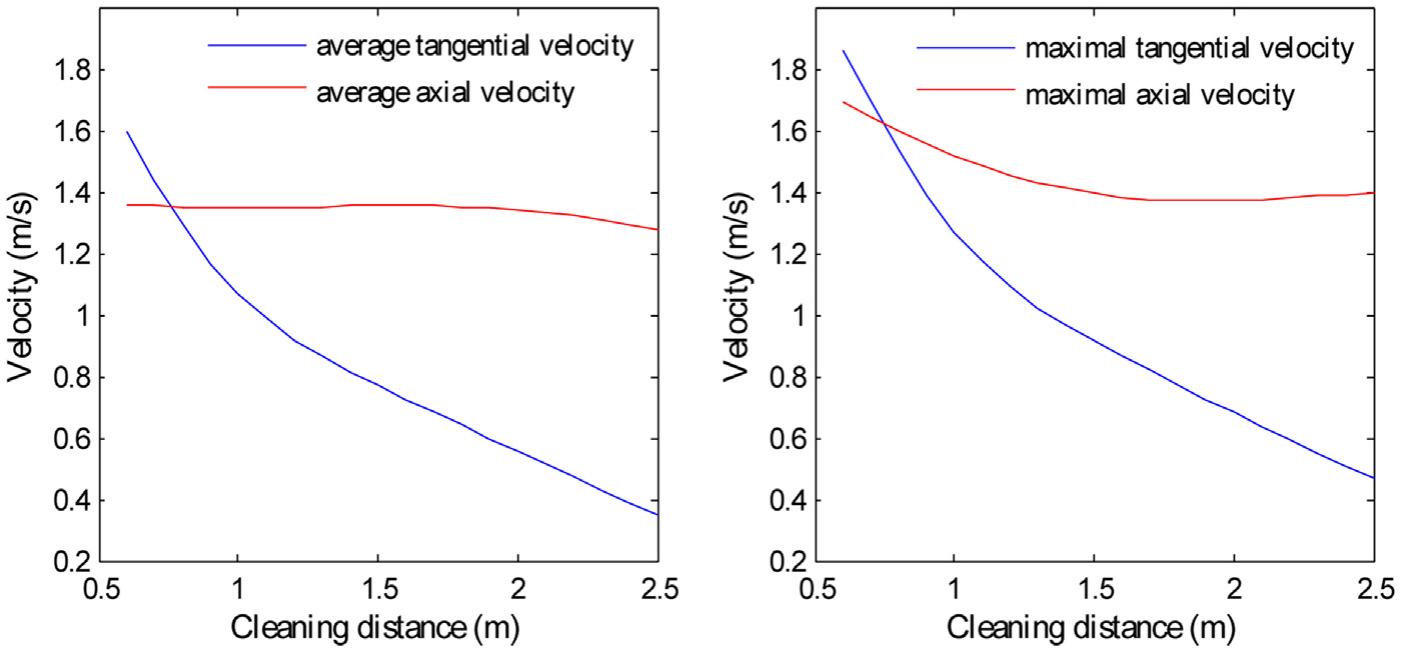
Distributions of V_t,avg_, V_a,avg_, V_t,max_ and V_a,max_ along the horizontal wellbore. No.3 nozzle assembly; flow rate 15.4 L/s; ambient pressure 35 MPa; ambient temperature is 363 K; jet temperature is 373 K.

The V_t,avg_, V_a,avg_, V_t,max_ and V_a,max_ distributions along the wellbore are shown in Fig 24. V_a,max_ decreases from the cleaning distance of 0.6 m to 2.0 m, which agrees with the simulation results. However, V_a,max_ has a slight increase when the cleaning distance is larger than 2.0 m. This may be because no sample data have a cleaning distance larger than 2.0 m. The velocities beyond 2.0 m are derived only through interpolation. Nevertheless, apart from V_a,max_, the other three curves have reasonable good decreasing shapes. The outcome may well be inaccurate if the input has some variables that are far from the simulation ranges. So it is suggested to use the model with inputs whose variables are within the simulation ranges or, less restrictively, not too far from the simulation ranges.

In addition, the other operation parameters such as nozzle assembly and jet temperature, if not fixed, can be optimized as well. The outcome will be the optimal set of operation parameters based on CSS. In the same case, the best nozzle assembly is No.1, and the best jet temperature is 353 K, with a minimal flow rate equal to 14.3 L/s. Compared to the previous result, the optimization of the nozzle assembly and jet temperature successfully reduces the minimum requirement of the flow rate from 15.4 L/s to 14.3 L/s, which can further reduce the cost of the pump and energy. It is proven that the BP-ANN optimization model is valid for optimizing single and multiple operation parameters based on CSS.

**Fig 25:**
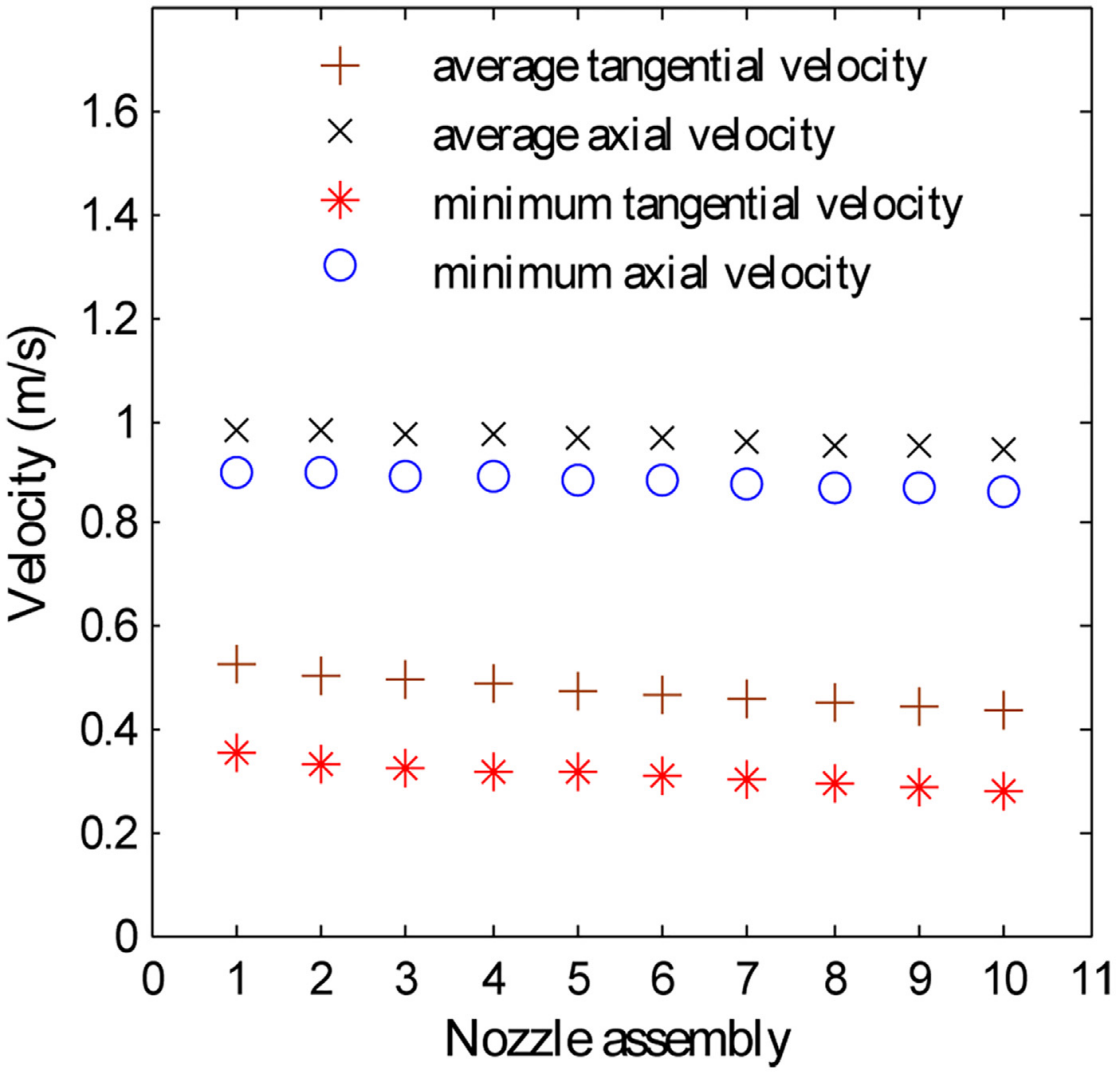
V_t,avg_ and V_a,avg_ with respect to different nozzle assemblies. Flow rate 12 L/s; ambient pressure 25.2 MPa; ambient temperature is 336.8 K; jet temperature is 360 K; cleaning distance 1.7 m.

*Case 2*. *LOS*. Suppose that for a horizontal well that requires a SC-CO_2_ helical cleanout, the TVD is 2300 m; the geothermal gradient is 1.6°C/100 m; the surface temperature is 27°C; the pressure gradient is 1.1 MPa/100 m; the jet temperature is 360 K; the cleaning distance is 1.7 m; a 12 L/s pump is the only pump available; all 10 nozzle assemblies are available; V_tc_ is 0.3 m/s; and V_ac_ is 0.8 m/s. Hydraulic losses are ignored for simplification. By recalling the BP-ANN model and programming, the V_t,min_, V_a,min_, V_t,avg_ and V_a,avg_ of each nozzle assembly at a critical cleaning distance of 1.7 m are acquired, as shown in Fig 25. The No.1–No.6 nozzle assemblies have V_t,min_ values that are larger than V_tc_, so they are the suitable nozzle assemblies that can satisfy the critical velocity requirement. Furthermore, it can be confirmed that the best nozzle assembly is the No.1 assembly.

Parameter optimizations using the BP-ANN model based on CSS and LOS are successfully performed. The constraint of V_tc_ and V_ac_ plays a key role in determining applicable operation parameters. The two strategies discussed are important but are not the only applications of the BP-ANN model. Another potential application of the BP-ANN model is to find the optimal traveling speed of the tool when performing wiper tripping in cleanout. The application can be further extended to cleanouts in inclined and vertical wells with various flushing fluids if cases that consider deviation angle are provided.

## 4. Conclusion

In this paper, CFD simulation is successfully applied to analyze the characteristics of SC-CO_2_ helical flow and sand sweeping efficiency in terms of V_t_ and V_a_. Comparisons are made between SC-CO_2_ helical flow and water helical flow. The influences of nozzle assembly, flow rate, ambient pressure, ambient temperature and jet temperature are obtained. The simulation data are then processed by BP-ANN, and the outcome model is able to optimize operation parameters in different situations. In all, the following conclusions can be drawn.

1. Compared to water helical flow cleanout, SC-CO_2_ helical flow cleanout, which can stimulate production, is more suitable to be applied in water-sensitive, low-permeability and conventional reservoirs.
2. In a horizontal annulus, the SC-CO_2_ helical flow field has more evenly radial distributions of V_a_ and smaller V_t_ due to its low viscosity. The nozzle assembly plays a major role in determining the characteristics of SC-CO_2_ helical flow. If other operation parameters are fixed, the suspending ability of SC-CO_2_ helical flow obviously improves with increasing lateral nozzle size and decreasing rear and forward nozzle size. For the simulated situations in this paper, the optimal nozzle assembly is identified as the No.1 assembly, whose rear, lateral and forward nozzles are 3 mm, 4 mm and 4 mm, respectively.
3. At certain cleaning distances, V_t_ and V_a_ improve linearly with higher ambient temperature or lower jet temperature. A significant discovery on the influence of temperature is that V_t_ and V_a_ depend on the difference between the ambient and jet temperatures, not on these two variables independently.
4. BP-ANN is successfully applied to match the operation parameters with V_t_ and V_a_ at various axial and radial positions. The outcome model is quite accurate, with R^2^ almost equal to 1. SVM is less efficient and accurate than BP-ANN for this problem. It is able to predict V_t_ and V_a_ precisely even if new combinations of operation parameters are provided. However, it is suggested that the model be used with input parameters that are within the simulation ranges, or at least not too far from the simulation ranges, for the purpose of veracity.
5. It is found that the BP-ANN optimization model is valid for optimizing single and multiple operation parameters. Two strategies are presented: the cost-saving strategy and the local optimal strategy. For the two hypothetical cases, optimizations based on the CSS and LOS strategies are performed successfully, which indicates the robustness of the BP-ANN optimization model. There is enough evidence to claim that the BP-ANN optimization model will work very well with field data.

## Supporting Information

S1 Dataset Axial and tangential velocities with respect to different ambient pressures. (XLS)

S2 Dataset Axial and tangential velocities with respect to different ambient temperatures. (XLS)

S3 Dataset Axial and tangential velocities with respect to different flow rate. (XLSX)

S4 Dataset Axial and tangential velocities with respect to different jet temperatures. (XLS)

S5 Dataset Axial and tangential velocities with respect to different nozzle assemblies. (XLSX)
